# Young Adults and Alcohol-Associated Liver Cancer: Incidence and Death from 2000 to 2021

**DOI:** 10.3390/cancers17040609

**Published:** 2025-02-11

**Authors:** Pojsakorn Danpanichkul, Yanfang Pang, Luis Antonio Diaz, Juan Pablo Arab, Soo Young Hwang, Disatorn Dejvajara, Varshini Suresh, Thanathip Suenghataiphorn, Chalothorn Wannaphut, Kanokphong Suparan, Karn Wijarnpreecha, Hyunseok Kim, Donghee Kim, Amit G. Singal, Ju Dong Yang

**Affiliations:** 1 Department of Internal Medicine, Texas Tech University Health Sciences Center, Lubbock, TX 79409, USA; 2 Affiliated Hospital of Youjiang Medical University for Nationalities, Baise 533099, China; 3 National Immunological Laboratory of Traditional Chinese Medicine, Baise 533000, China; 4 Center for Medical Laboratory Science, Affiliated Hospital of Youjiang Medical University for Nationalities, Baise 533099, China; 5 Department of Microbiology, Faculty of Medicine, Chiang Mai University, Chiang Mai 50200, Thailand; 6 Metabolic-Dysfunction Associated Steatotic Liver Disease Research Center, Division of Gastroenterology and Hepatology, University of California San Diego, San Diego, CA 92037, USA; 7 Departamento de Gastroenterologia, Escuela de Medicina, Pontificia Universidad Catolica de Chile, Santiago 8320165, Chile; 8 Observatorio Multicéntrico de Enfermedades Gastrointestinales, OMEGA, Santiago, Chile; 9 Division of Gastroenterology, Hepatology, and Nutrition, Department of Internal Medicine, Virginia Commonwealth University School of Medicine, Richmond, VA 23298, USA; 10 Department of Internal Medicine, University of Maryland Medical Center, Midtown Campus, Baltimore, MD 21201, USA; 11 Faculty of Medicine, Chiang Mai University, Chiang Mai 50200, Thailand; 12 School of Medicine, Texas Tech University Health Sciences Center, Lubbock, TX 79409, USA; 13 Department of Internal Medicine, Griffin Hospital, Derby, CT 06418, USA; 14 Department of Medicine, John A. Burns School of Medicine, University of Hawaii, Honolulu, HI 96813, USA; 15 Immunology Unit, Department of Microbiology, Faculty of Medicine, Chiang Mai University, Chiang Mai 50200, Thailand; 16 Division of Gastroenterology and Hepatology, Department of Medicine, University of Arizona College of Medicine, Phoenix, AZ 85004, USA; 17 Department of Internal Medicine, Banner University Medical Center, Phoenix, AZ 85004, USA; 18 BIO5 Institute, University of Arizona College of Medicine-Phoenix, Phoenix, AZ 85004, USA; 19 Karsh Division of Gastroenterology and Hepatology, Comprehensive Transplant Center, Samuel Oschin Comprehensive Cancer Institute, Cedars-Sinai Medical Center, Los Angeles, CA 90048, USA; 20 Division of Gastroenterology and Hepatology, Stanford University School of Medicine, Stanford, CA 94063, USA; 21 Division of Digestive and Liver Diseases, University of Texas Southwestern Medical Center, Dallas, TX 75390, USA

**Keywords:** addiction, alcohol-associated liver disease, cancer, oncology, public health

## Abstract

Background/Objectives: The incidence and mortality of early-onset cancers have been rising in recent decades. While epidemiological studies have examined various types of cancer, updated global data on alcohol-attributable primary liver cancer (PLC) in young adults remains limited. Methods: This study utilized data from the Global Burden of Disease (GBD) study spanning 2000 to 2021 to evaluate the age-standardized incidence, mortality, and corresponding rates of alcohol-attributable PLC among young adults aged 15–49 years. Results: In 2021, there were 8290 incidence cases and 6590 deaths from alcohol-attributable PLC in young adults. The age-standardized incidence rate has increased in Europe (annual percent change [APC]: 0.44%, 95%CI 0.35 to 0.54%), Southeast Asia (APC: 0.40%, 95% CI 0.37 to 0.44%), and the Western Pacific region (APC: 0.65%, 95% CI 0.44 to 0.86%). In 2021, alcohol-attributable PLC in young adults represented 11% (+2% from 2000) of incident cases and 11% of (+2% from 2000) deaths among all PLC in young adults. About half of the countries showed an increase in the age-standardized incidence rate of alcohol-attributable PLC among young adults between 2000 and 2021. Conclusions: The incidence rate of alcohol-attributable liver cancer in young adults has shifted significantly over the past two decades, with notable increases in Europe and the Asia–Pacific region. This trend underscores the need for global strategies to address the rising prevalence of alcohol use disorder and alcohol-associated liver disease and their impact on young adults.

## Simple Summary

Alcohol, a known hepatocarcinogen, has increasingly contributed to the risk of primary liver cancer development in young adults over the past two decades. In 2021, more than 8000 young adults were diagnosed with alcohol-attributable liver cancer, and approximately 6500 died. Over half of all countries experienced a rise in incidences during this period, with the largest increases observed in Europe, the Asia–Pacific region, and countries with low–middle and middle sociodemographic indices. This growing burden of alcohol-attributed liver cancers in young adults underscores the need for urgent strategic plans from global and regional taskforces to enhance health outcomes and mitigate socioeconomic impacts.

## 1. Introduction

Cancer has a major impact across the world. According to Global Cancer Statistics 2020, 19.3 million new cases emerged, with almost 10.0 million cancer deaths worldwide in 2020 [1]. Although cancer has traditionally been considered a disease caused by aging, the incidence of early-onset cancers is increasing [2,3,4]. Early-onset cancer is defined as cancer occurring in individuals under the age of 50 [5,6]. This form of cancer is distinct due to different risk factors, tumor biology, and survivorship compared to cancer in general populations.

Primary liver cancer (PLC) is the third leading cause of cancer-related death globally and continues to rise in number among multiple age strata [1]. In contrast to other cancers, the incidence of overall PLC in young adults has decreased in recent decades [1,7]. This is likely due to advancements in managing chronic viral hepatitis, including widespread vaccination and medications against the hepatitis B and hepatitis C viruses to a lesser extent. However, recent decades have also seen a rising wave of alcohol consumption and alcohol-associated liver disease (ALD) among younger adults. Alcohol is the most common cause of liver cancer, accounting for 32% to 45% of liver cancer incidences [8,9]. With lower surveillance rates and more advanced staging when diagnosed compared with other etiologies of PLC, the absolute burden of alcohol-related liver cancer has been increasing [10].

Given the increased prevalence of ALD in young adults, coupled with the rising trend of cancer in this age group, these factors may have significantly influenced the recent global epidemiology of alcohol-attributable PLC in young adults [11]. Updated data characterizing the burden of alcohol-attributable PLC at the global, regional, national, sex, and sociodemographic levels are crucial.

This study aimed to investigate temporal trends in alcohol-attributable PLC incidence and deaths across 204 countries and territories in young adults, using the latest data from the Global Burden of Disease (GBD) Study 2021 [12,13,14].

## 2. Materials and Methods

### 2.1. Data Source

This study utilized data on alcohol-attributable PLC incidence and death from 2000 to 2021, extracted from the GBD 2021 dataset [15]. Differences in the rates of incidence and death for alcohol-attributable PLC were assessed across sex, region, and country. Data were obtained through the Global Health Data Exchange (GHDx) query tool, a resource maintained by the Institute for Health Metrics and Evaluation (http://ghdx.healthdata.org/gbd-results-tool, accessed on 10 November 2024), which provides annual counts and age-standardized rates (ASRs) for alcohol-attributable PLC incidence and mortality, classified by sex, region, and country [12]. This was accessed on 10 November 2024.

### 2.2. Estimation Methods

The estimation methods for GBD 2021 and the approach for calculating the burden of alcohol-attributable PLC are detailed in prior GBD publications and Supplementary Material S1 [16,17]. Standardized coding was employed to ensure the uniform identification of alcohol-attributable PLC across diverse countries and regions. Similarly to other early-onset cancers, in this study, alcohol-associated PLC in young adults is defined as PLC diagnosed in individuals aged 15–49 years. Countries were classified by development level using the sociodemographic index (SDI), which incorporates total fertility rate, average educational attainment, and income per capita (Supplementary Material S2). SDI categories include high (above the 80th percentile), high–middle (60th–79th percentiles), middle (40th–59th percentiles), low–middle (20th–39th percentiles), and low (below the 20th percentile). The burden of alcohol-attributable PLC was further grouped into six regions based on the World Health Organization (WHO) classification: Africa, Eastern Mediterranean, Europe, the Americas, Southeast Asia, and the Western Pacific. Comparisons of alcohol-attributable PLC burden with other etiologies were also conducted.

### 2.3. Statistical Analysis

To thoroughly capture data variability and uncertainty in statistical modeling, each incidence and mortality estimate in the study was reported with 95% uncertainty intervals (UIs), determined by the 2.5th and 97.5th ranked values from 1000 draws of the posterior distribution. This method provides a nuanced understanding of data variability. ASRs per 100,000 population were calculated following the GBD 2021 population estimates, allowing for consistent comparisons across populations and timeframes. This study also computed the annual percent change (APC) in ASRs, with 95% confidence intervals (CIs), to assess temporal trends: an APC with a positive value and *p* < 0.05 indicates an upward trend, while a negative APC with *p* < 0.05 indicates a downward trend; changes with *p* ≥ 0.05 are deemed insignificant. The Joinpoint regression program (version 4.9.1.0) from the National Cancer Institute was used for this analysis. Additionally, this study calculated the proportion of young adults with alcohol-attributable PLC relative to all PLC cases in this age group.

## 3. Results

### 3.1. Global Burden of Alcohol-Associated Primary Liver Cancer in Young Adults in 2021

**Figure 1:**
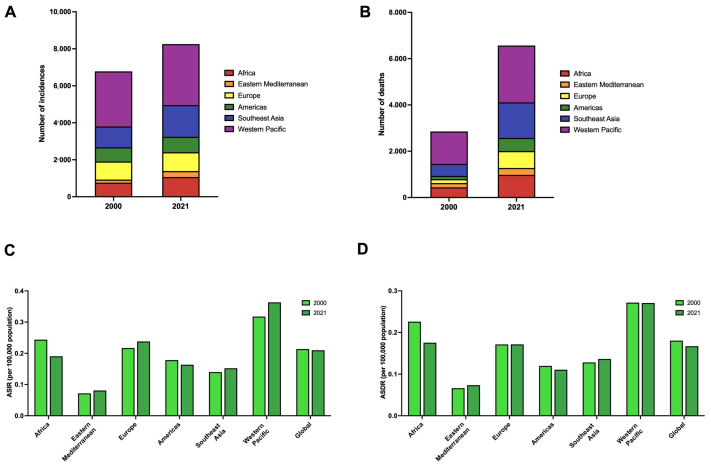
The trend in the burden of alcohol-attributable primary liver cancer in young adults, stratified by World Health Organization Region. (**A**) The number of incidences of alcohol-attributable primary liver cancer in young adults in 2000 and 2021, stratified by World Health Organization Region. (**B**) The number of deaths from alcohol-attributable primary liver cancer in young adults in 2000 and 2021, stratified by World Health Organization Region. (**C**) Age-standardized incidence rates of alcohol-attributable primary liver cancer in young adults in 2000 and 2021 by World Health Organization Region. (**D**) Age-standardized death rates for alcohol-attributable primary liver cancer in young adults in 2000 and 2021 by World Health Organization Region. Legend: ASDR: age-standardized death rate; ASIR: age-standardized incidence rate.

Globally, in 2021, the number of alcohol-attributable PLC incidences and deaths in young adults was estimated to be 8290 (95% UI: 5770 to 11,340) incident cases and 6590 (95% UI: 4570 to 9040) deaths (Table 1 and Figure 1A,B). In 2021, the age-standardized incidence rate (ASIR) and age-standardized death rate (ASDR) were 0.21 (95% UI: 0.15 to 0.29) per 100,000 and 0.17 (95% UI: 0.12 to 0.23) per 100,000, respectively (Table 1 and Figure 1C,D). The ASIR (APC: −0.10%; 95% CI: −0.18 to −0.03%) and ASDR (APC: −0.38%; 95% CI: 0.49 to −0.27%) decreased (Table 1).

**Figure 2:**
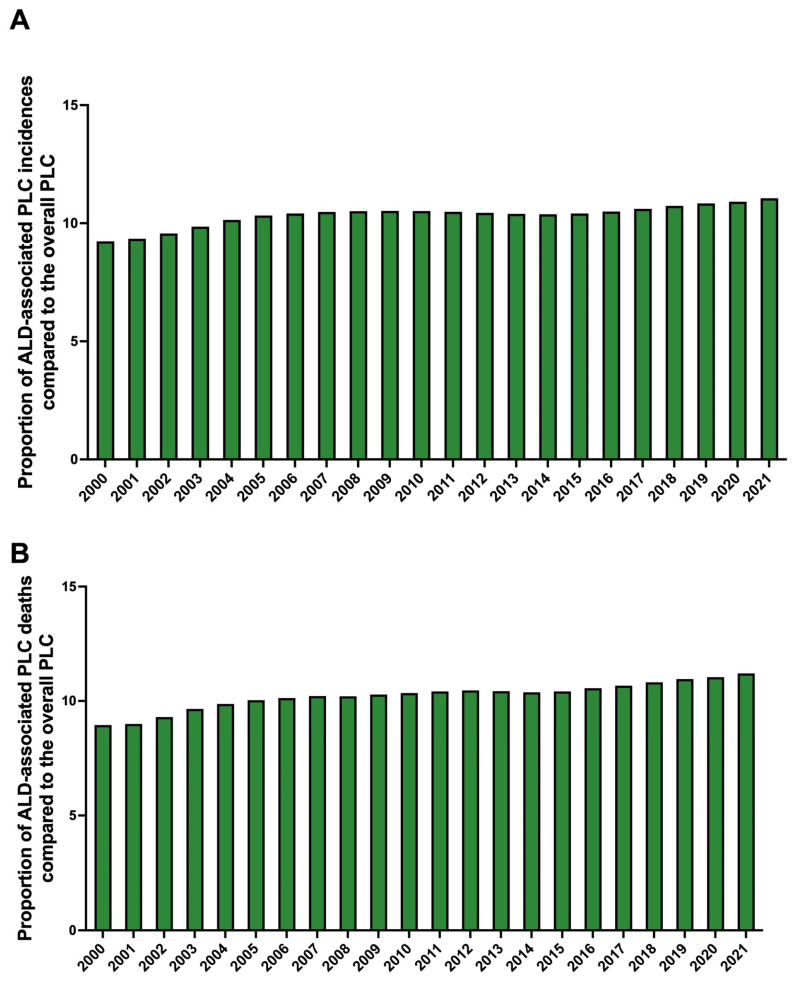
The contribution of alcohol-attributable primary liver cancer compared to the overall rates of primary liver cancer in young adults from 2000 to 2021. (**A**) The contribution of the incidence of alcohol-attributable primary liver cancer compared to the overall incidence of primary liver cancer in young adults from 2000 to 2021. (**B**) Contribution of death from alcohol-attributable primary liver cancer compared to the overall rate of death from primary liver cancer in young adults from 2000 to 2021. Legend: ALD: alcohol-associated liver disease.

In 2021, alcohol-attributable PLC in young adults represented 11% (+2% since 2000) of incident cases and 11% (+2% since 2000) of deaths among all causes of PLC in young adults (Figure 2A,B).

### 3.2. The Burden of Alcohol-Attributable Primary Liver Cancer in Young Adults by Sex

**Figure 3:**
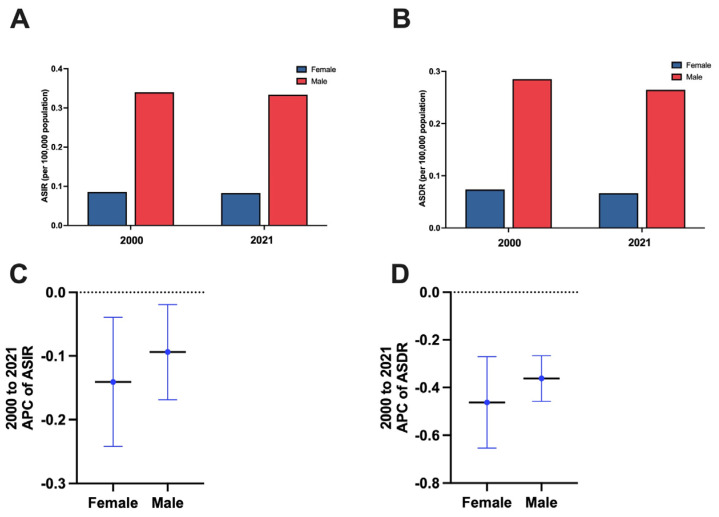
The trend in the burden of alcohol-attributable primary liver cancer in young adults, stratified by sex. (**A**) Age-standardized incidence rates of alcohol-attributable primary liver cancer in young adults in 2021 by sex. (**B**) Age-standardized death rates for alcohol-attributable primary liver cancer in young adults in 2021 by sex. (**C**) Annual percent change from 2000 to 2021 in age-standardized incidence rates attributable to alcohol-associated primary liver cancer in young adults by sex. (**D**) Annual percent change from 2000 to 2021 in age-standardized death rates attributable to alcohol-associated primary liver cancer in young adults by sex. Legend: ASDR: age-standardized death rate; ASIR: age-standardized incidence rate; APC: annual percent change.

The incidence and death rates were higher in males compared to females. In 2021, the number of incidents of alcohol-attributable PLC cases in young females was 1610 (95% UI: 1120 to 2250), and there were 1300 (95% UI: 900 to 1830) deaths. In young males, there were 6670 (95% UI: 4630 to 9060) incident cases and 5290 (95% UI: 3660 to 7270) deaths (Table 1). The ASIR and ASDR of young females were 0.08 (95% UI: 0.06 to 0.12) and 0.07 (95% UI: 0.05 to 0.09) per 100,000 population, respectively. In young males, the ASIR and ASDR of alcohol-attributable PLC were 0.33 (95% UI: 0.23 to 0.45) and 0.26 (95% UI: 0.18 to 0.36) per 100,000 population (Figure 3A,B). From 2000 to 2021, the ASIR and ASDR decreased in females. Meanwhile, in males, the ASIR (APC: −0.09, −0.17 to −0.02%) and ASDR (−0.36%; 95% CI: −0.46 to −0.27%) decreased (Figure 3C,D).

### 3.3. The Burden of Alcohol-Attributable Primary Liver Cancer in Young Adults, According to World Health Organization Region

In 2021, the Western Pacific region had the highest alcohol-attributable PLC incidence (n = 3300) and number of deaths (n = 2460) in young adults (Figure 1A,B). The ASIR and ASDR were highest in the Western Pacific region, with values of 0.36 (95% UI: 0.24 to 0.52) and 0.27 (95% UI: 0.18 to 0.39) per 100,000 population (Figure 1C,D). Between 2000 and 2021, the ASIR increased in the Eastern Mediterranean region (APC: 0.57%; 95% CI: 0.53 to 0.60%), Europe (APC: 0.44%; 95% CI: 0.35 to 0.54%), Southeast Asia (APC: 0.40%; 95% CI: 0.37 to 0.44%), and the Western Pacific region (APC: 0.65%; 95% CI: 0.44 to 0.86%) (Table 1).

### 3.4. The Burden of Alcohol-Attributable Primary Liver Cancer in Young Adults, According to Sociodemographic Index

**Figure 4:**
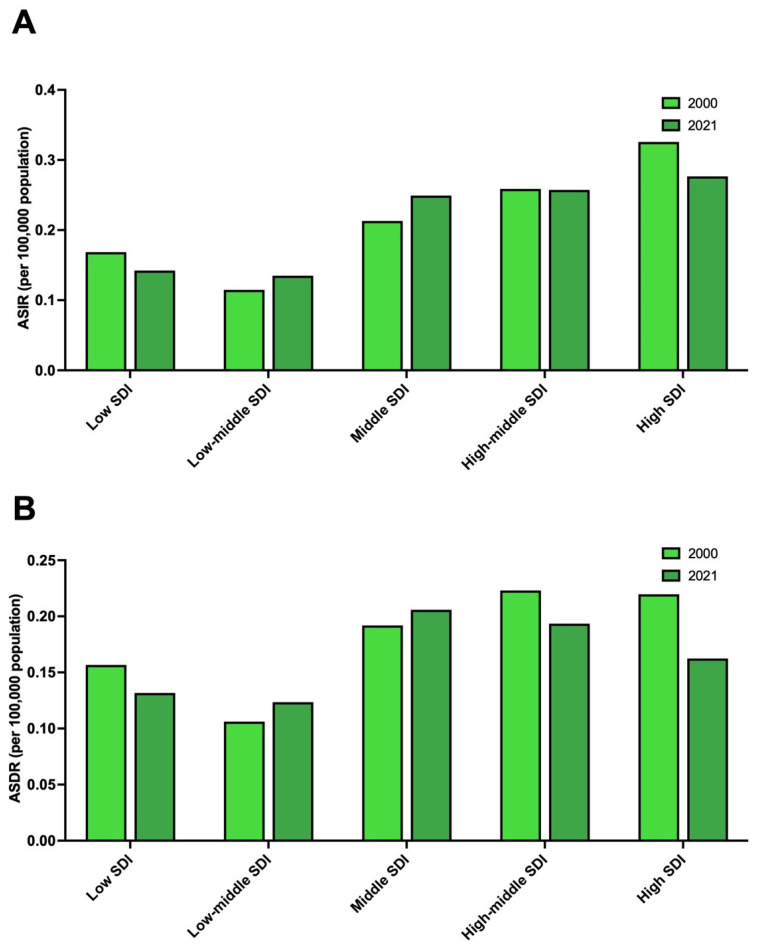
The trend in the burden of alcohol-attributable primary liver cancer in young adults, stratified by sociodemographic index. (**A**) Age-standardized incidence rates attributable to alcohol-attributable primary liver cancer in young adults in 2000 and 2021 by sociodemographic index. (**B**) Age-standardized death rates attributable to alcohol-attributable primary liver cancer in young adults in 2000 and 2021 by sociodemographic index. Legend: ASDR: age-standardized death rate; ASIR: age-standardized incidence rate; SDI: sociodemographic index.

In 2021, the highest frequencies of alcohol-attributable PLC cases (n = 3130) and deaths (n = 2580) in young adults were observed in middle-SDI countries (Table 1). The most pronounced ASIR per 100,000 population was also observed in high-SDI countries, with a value of 0.28 (95% UI: 0.21 to 0.37) per 100,000 population (Figure 4A). Contrarily, the ASDR was highest in middle-SDI countries, with an ASDR of 0.21 (95% UI: 0.14 to 0.29) (Figure 4B). Between 2000 and 2021, the ASIR underwent the greatest increase in low–middle- (APC: 0.76%; 95% CI: 0.73 to 0.79%) and middle- (APC: 0.72%; 95% CI: 0.58 to 0.87%) SDI countries (Table 1). The ASDR increased in low–middle- (APC: 0.70%; 95% CI: 0.48 to 0.93%) and middle- (APC: 0.36%; 95% CI: 0.18 to 0.54%) SDI countries (Table 1).

### 3.5. The Burden of Alcohol-Attributable Primary Liver Cancer in Young Adults by Country

**Figure 5:**
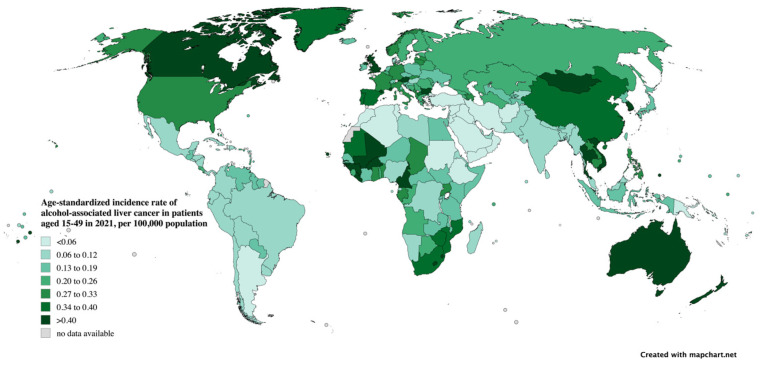
Age-standardized incidence rates in 2021 of alcohol-attributable primary liver cancer in young adults in 2021 by country.

The countries with the highest ASIRs from alcohol-associated PLC are Mongolia, the Kingdom of Thailand, and the Republic of the Gambia. Mongolia has an ASIR of 2.75 (95% UI 1.42 to 4.86), the Kingdom of Thailand has an ASIR of 1.16 (95% UI 0.61 to 1.97), and the Republic of the Gambia has an ASIR of 1.04 (95% UI 0.48 to 2.00) per 100,000 population, respectively (Figure 5 and Supplementary Table S1). Approximately half of the countries and territories exhibited an uptrend in ASIR due to alcohol-associated PLC, with Poland (APC: 6.58%, 95% CI 6.30 to 6.85%), Cook Island (APC: 4.85%, 95% CI 4.72 to 4.98%), and Uruguay (APC: 4.64%, 95% CI 4.31 to 4.96%) having the largest increases (Supplementary Table S1).

### 3.6. The Burden of Alcohol-Associated Cirrhosis and Chronic Liver Disease

Globally, in 2021, the prevalence of alcohol-associated cirrhosis and chronic liver disease in young adults was estimated to be 1.02 million cases (Supplementary Table S2). In 2021, the age-standardized prevalence rate was 25.79 (95% UI: 19.87 to 32.67) per 100,000 population. The ASPR (APC: −0.13%; 95% CI: −0.17 to −0.10%) decreased from 2000 to 2021 (Supplementary Table S2). Regionally, the ASPR increased in the Eastern Mediterranean region (APC: 0.21%; 95% CI: 0.16 to 0.27%), Europe (APC: 0.17%; 95% CI: 0.02 to 0.32%), Southeast Asia (APC: 0.45%; 95% CI: 0.42 to 0.49%), and the Western Pacific region (APC: 0.56%; 95% CI: 0.50 to 0.61%). Regarding the SDI, the ASPR from alcohol-associated cirrhosis and chronic liver disease in young adults increased in low–middle- (APC: 0.25%; 95% CI: 0.23 to 0.28%), middle- (APC: 0.58%; 95% CI: 0.53 to 0.63%), and high–middle-SDI countries (APC: 0.42%; 95% CI: 0.27 to 0.57%) (Supplementary Table S2).

## 4. Discussion

Our findings highlight a decline in the incidence and mortality of alcohol-attributable PLC in young adults over the past two decades. However, significant regional differences persisted, with incidence rates rising in four of the six regions, including the Eastern Mediterranean, Europe, Southeast Asia, and the Western Pacific. According to the SDI classification, low–middle- and middle-SDI countries experienced increases in alcohol-attributable PLC incidence among young adults. Additionally, alcohol-attributable PLC has contributed an increasing share of the global burden, accounting for 11% of both new cases and deaths in 2021. Over the last 20 years, nearly half of the countries showed a rising trend in alcohol-attributable PLC among young adults.

While the incidence and death rates per 100,000 population for alcohol-attributable PLC in young adults were declining before, the total number of cases and deaths increased between 2000 and 2021. This could be due to a delay in the onset of alcohol-attributable PLC and related deaths in this younger population or the extended time required for diagnosis, as alcohol-attributable PLC is often detected at more advanced stages compared to PLC from other causes [10,18,19,20]. Other contributing factors may include comorbidities that exacerbate the disease, smoking, gut dysbiosis, and genetic susceptibility [21,22]. In fact, recent evidence has shown that individuals with metabolic dysfunction and alcohol-associated liver disease (MetALD) exhibit a higher risk of hepatocellular carcinoma than those with ALD [23,24,25]. Particularly in patients with diabetes, insulin resistance and hyperinsulinemia accelerate hepatocarcinogenesis [26,27,28,29]. However, GBD data do not currently capture the incidence and mortality of MetALD. Future iterations of the GBD should incorporate these metabolic factors and update the nomenclature for steatotic liver disease [30,31,32,33].

Our study found that the incidence rate of alcohol-attributable PLC disproportionately increased in low–middle- and middle-SDI countries, highlighting their growing contribution to the global burden of alcohol-attributable PLC. Factors such as rising purchasing power and the lack of effective public policies likely drive the increase in ALD and PLC among young adults in these regions. In general, several ecological studies have suggested the effectiveness of public health policies in reducing alcohol-related harm [34]. Policymakers should prioritize strengthening alcohol regulations, particularly targeting adolescents and young adults [35,36]. Public education and awareness campaigns are vital to promote responsible alcohol consumption, while early screening and brief interventions can help identify and address hazardous drinking patterns and alcohol use disorder [37,38,39]. Furthermore, healthcare workers play a crucial role in implementing these strategies, particularly in addressing challenges such as collecting alcohol use histories, quantifying consumption, and closely monitoring alcohol use. The obtained information would help policymakers specifically address the root cause of ALD and PLC in young adults. Together, these measures are crucial to reduce the burden of alcohol-related harm and mitigate the growing prevalence of early-onset cancers.

This study has several acknowledged limitations, many of which are common challenges inherent to GBD analyses and stem from the availability and quality of primary data, often dependent on the efficacy of vital registration systems within individual countries [40,41,42]. The GBD estimation methodology may contribute to an underestimation of mortality, particularly in resource-limited countries, due to the lack of high-quality data and underreported risk factors influenced by the stigma around alcohol consumption. Notably, the GBD does not detail how alcohol contributes to liver pathologies across the spectrum [43,44]. As a result, epidemiological metrics for alcohol-associated hepatitis and alcohol-induced acute-on-chronic liver failure are not available, limiting our understanding of how alcohol influences epidemiological changes [45,46,47,48,49]. Additionally, the GBD is unable to account for other relevant factors, such as the interactions between alcohol use, viral hepatitis, and risk factors [50,51,52,53]. Moreover, the GBD analytical framework is also limited in attributing a single cause to a specific outcome, excluding the potential co-variation in other contributing risk factors [50,54,55]. Future modeling studies should aim to incorporate additional comorbidities and determinants of health, such as ethnicity and socioeconomic and cultural factors.

## 5. Conclusions

Over the past two decades, the incidence rate of alcohol-attributable PLC in young adults has changed significantly, with marked increases observed in Europe and the Asia–Pacific region. This highlights the pressing need for global strategies to tackle the growing prevalence of alcohol use disorder, ALD, and its effects on young adults.

## Figures and Tables

**Table 1:** Incidence, death, and age-standardized rates of patients with alcohol-attributable primary liver cancer in young adults in 2021 and changes from 2000 to 2021.

	Incidence				Death			
	2021 Number (95% UI)	2021 Age-Standardized Incidence Rate (95% UI)	2000 to 2021 Annual Percent Change (95% CI)	*p*	2021 Number (95% UI)	2021 Age-Standardized Death Rate (95% UI)	2000 to 2021 Annual Percent Change (95% CI)	*p*
Both	8290 (5770 to 11,340)	0.21 (0.15 to 0.29)	−0.1 (−0.18 to −0.03)	0.008	6590 (4570 to 9040)	0.17 (0.12 to 0.23)	−0.38 (−0.49 to −0.27)	<0.001
**By Sex**								
Female	1610 (1120 to 2250)	0.08 (0.06 to 0.12)	−0.14 (−0.24 to −0.04)	0.007	1300 (900 to 1830)	0.07 (0.05 to 0.09)	−0.46 (−0.65 to −0.27)	<0.001
Male	6670 (4630 to 9060)	0.33 (0.23 to 0.45)	−0.09 (−0.17 to −0.02)	0.015	5290 (3660 to 7270)	0.26 (0.18 to 0.36)	−0.36 (−0.46 to −0.27)	<0.001
**By WHO Region**								
Africa	1070 (630 to 1670)	0.19 (0.11 to 0.3)	−1.18 (−1.34 to −1.02)	<0.001	980 (580 to 1540)	0.18 (0.1 to 0.27)	−1.2 (−1.29 to −1.11)	<0.001
Eastern Mediterranean	320 (210 to 480)	0.08 (0.05 to 0.12)	0.57 (0.53 to 0.6)	<0.001	290 (190 to 430)	0.07 (0.05 to 0.11)	0.45 (0.09 to 0.8)	0.013
Europe	1020 (750 to 1350)	0.24 (0.17 to 0.32)	0.44 (0.35 to 0.54)	<0.001	730 (540 to 980)	0.17 (0.13 to 0.23)	0.03 (−0.46 to 0.53)	0.892
Region of the Americas	840 (660 to 1050)	0.16 (0.13 to 0.2)	−0.4 (−0.52 to −0.27)	<0.001	570 (440 to 720)	0.11 (0.09 to 0.14)	−0.41 (−0.71 to −0.11)	0.008
Southeast Asia	1710 (1210 to 2340)	0.15 (0.11 to 0.21)	0.4 (0.37 to 0.44)	<0.001	1540 (1080 to 2100)	0.14 (0.1 to 0.19)	0.3 (0.01 to 0.59)	0.046
Western Pacific	3300 (2210 to 4690)	0.36 (0.24 to 0.52)	0.65 (0.44 to 0.86)	<0.001	2460 (1630 to 3540)	0.27 (0.18 to 0.39)	−0.1 (−0.57 to 0.37)	0.674
**By SDI**								
Low SDI	770 (450 to 1260)	0.14 (0.08 to 0.23)	−0.82 (−0.92 to −0.72)	<0.001	710 (420 to 1180)	0.13 (0.08 to 0.22)	−0.86 (−1.03 to −0.7)	<0.001
Low–Middle SDI	1370 (930 to 1930)	0.13 (0.09 to 0.19)	0.76 (0.73 to 0.79)	<0.001	1250 (850 to 1770)	0.12 (0.08 to 0.17)	0.7 (0.48 to 0.93)	<0.001
Middle SDI	3130 (2140 to 4330)	0.25 (0.17 to 0.34)	0.72 (0.58 to 0.87)	<0.001	2580 (1770 to 3580)	0.21 (0.14 to 0.29)	0.36 (0.18 to 0.54)	<0.001
High–Middle SDI	1620 (1120 to 2180)	0.26 (0.18 to 0.35)	−0.11 (−0.31 to 0.1)	0.311	1220 (840 to 1650)	0.19 (0.13 to 0.26)	−0.75 (−1.24 to −0.26)	0.003
High SDI	1390 (1040 to 1840)	0.28 (0.21 to 0.37)	−0.78 (−0.87 to −0.69)	<0.001	820 (610 to 1090)	0.16 (0.12 to 0.22)	−1.47 (−1.71 to −1.23)	<0.001

Abbreviations: CI: confidence interval; SDI: sociodemographic index; UI: uncertainty interval; WHO: World Health Organization.

## Data Availability

The Global Burden of Disease Study 2021 offers comprehensive data on the burden of diseases and risk factors across 204 countries and territories. Access to these data is provided by the GlobalHealth Data Exchange query tool (http://ghdx.healthdata.org/gbd-results-tool, accessed on 10 November 2024), which the Institute for Health Metrics and Evaluation maintains.

## Supplementary Materials

The following supporting information can be downloaded at https://www.mdpi.com/article/10.3390/cancers17040609/s1: Material S1: Overview of Global Burden of Disease Methodology; Material S2: Sociodemographic index of national and subnational based on the GBD 2021 study. Table S1: Incidence and age-standardized rates of patients with alcohol-associated primary liver cancer in young adults in 2000 and 2021 and changes from 2000 to 2021. Table S2: Prevalence and age-standardized prevalence rates of patients with alcohol-associated cirrhosis and chronic liver disease in young adults in 2021 and changes from 2000 to 2021.
