# Supplementary material for: Young Adults and Alcohol-Associated Liver Cancer: Incidence and Death from 2000 to 2021
Source: Cancers (Basel). 2025 Feb 11;17(4):609. doi: 10.3390/cancers17040609 (PMC11853673; doi:10.3390/cancers17040609)
Supplement: Supplementary file 1 [file cancers-17-00609-s001.zip › cancers-3403797-supplementary.pdf]

Supplementary Material S1: Overview of Global Burden of Disease Methodology

1. Overview

The GBD 2021 estimates mortality and causes of mortality for diseases in 204 countries and territories using a standard methodological approach.

The GBD 2021 uses a variety of data input sources, including surveys, censuses, vital statistics, and other health-related data sources to estimate mortality rates. The input sources are available via the interactive citation tool using the Global Health Data Exchange (GHDx; http://ghdx.healthdata.org/). The user can view and access GHDx records for input sources and export a comma-separated value (CSV) file comprising metadata, citations, and information on where data were used in GBD. Citations for specific GBD components, causes and risks, and locations can be located via this tool. Moreover, the GBD allows the visualisation of its results online. All GBD 2021 online data visualisations are available at https://vizhub.healthdata.org/gbd-compare/, which offers results for all GBD health metrics. The core summary GBD 2021 results comprising mortality data can be obtained in tabular form with the GBD's data download tool, available at http://ghdx.healthdata.org/gbd-results-tool. Users must provide an email address, depending on download size, where a download location will be provided to them when the files are ready.

2. Causes of Death Database

Data sources for causes of death were retrieved from vital registration systems, verbal autopsies, and other surveillance systems from 2000-2021. The available data on the cause of death (CoD) are standardised based on the International Classification of Diseases (ICD) 9 and 10 code mapping and pooled into a single database used to generate cause-specific mortality estimates by sex, year, regional geography and age. The CoD database includes seven data sources, including vital registration (VR), verbal autopsy (VA), sibling history, and survey/census. There is no need to use any other data source in countries with complete VR systems. However, less than half of the global population has deaths captured in a VR system. Hence, for these countries with incomplete VR systems, vital statistics for causes of death may be supplemented with other data types[3]. Data inputs used to generate the estimates are found at http://ghdx.healthdata.org/gbd-2021/data-input-sources.

Most of the CoD data is VR data obtained from WHO Mortality Database, a summation of data submitted to WHO by individual countries. VR is obtained from country-specific mortality databases operated by official offices. Whenever possible, each cause is coded directly to the most detailed CoD, whilst cause codes in data tabulated by ICD are coded to aggregated cause groups. Many countries have adopted the ICD Tabulation lists. The ICD tabulation lists include the ICD-9 Basic Tabulation List, the ICD-10 Mortality Tabulation, the Russia Tabulation, and the India Medical Certification of Cause of Death. Sample registration systems are increasingly used in several countries, such as Indonesia and India. In those without VR systems, VA studies are a viable data source to inform CoD. Data are retrieved by trained interviewers who utilise a standardised questionnaire to ask relatives about the signs, symptoms, and demographic characteristics of recently deceased family members. CoD is assigned based on the answers to the questionnaires. Hence, VA data are highly heterogeneous as studies adopt different instruments, cause lists (from single causes to complete ICD-cause lists), methods for assigning CoD, recall periods, and age groups. Cultural differences may also play a part in affecting the interpretation of specific questions. When mapping to the GBD cause, CoD validity might be considered. VAs are likely less accurate for causes requiring medical certification, such as diabetes, than assigning CoD to road injury or homicide.

3. Steps in Data Input

Step 1: Standardise input data

Diverse formats such as mortality databases, literature reviews and reports were used to collect the input data for the cause of death (CoD) database. Usable data sources must demonstrate an apparent sample size of the number of deaths in the population and exhaustive cause lists. The data cleaning process remains complex and differs across data sources; for instance, minimal effort is needed to standardise the data into a consistent structure for VR microdata with location, age, sex, year, and ICD-coded cause of death. The data are subsequently assigned source identifiers to be linked to the GHDx and cited appropriately. Aggregate sex and age categories are then identified for sex-age splitting. Documentation from the source is reviewed to ensure that the population is representative of the location or only a subgroup of the population within that region. Diagnostics were also reviewed at the final stage to avoid sending cleaning errors downstream. All death totals were compared with the sum of cause-specific deaths to ensure that all observed deaths were accounted for and the sample size was complete.

The CoD in tabulated VR data were then compiled into aggregated groups, and some were mapped directly to GBD causes, while others were not informative and thus, cannot be mapped to them. The aggregated causes were mapped and split into ICD detail causes or targets based on the ICD groupings within the aggregated causes. The proportions of deaths from nearby countries with the super-region were used to fill in data gaps as they tended to have similar CoD trends. Global proportions were used for any cause and demographic group for which ICD-detail was lacking.

Step 2: Map to GBD cause list

GBD 2021 used 439 maps to ensure that the cause list from the input data matched the GBD cause list. Examples of the largest and most widely accepted maps used were those of the ICD9 and ICD10 VR data. The mapping process allowed for comparing various data sources across demographic groups.

Step 3: Split Age-sex groups

Some input sources, particularly the VA studies, reported death for a wide range of age groups with different intervals. These different age intervals were then mapped to the GBD standard set of age groups. When the input source contains death due to impossible causes for the given sex and age, it was then redistributed proportionally to all causes.

Step 4: Redistribute

To improve the comparability of the data for CoD, redistribution of uninformative codes, or garbage codes, is needed. Garbage codes to which deaths were assigned should not be considered as the underlying CoD (for example, "heart failure", "ill-defined cancer site"). For each redistribution package, we defined the "universe" of data as all deaths coded to either the package's garbage codes or the package's redistribution targets for each country, year, age, and sex. A regression was run separately for each target group and sex.

Step 5: Correct post-redistribution problems

In this step, data were checked to ensure that the cause list at this point is reasonable given the original data source and how the CoD was assigned. There were two primary corrections that were applied – 1) any cause that is purely an artifact of the redistribution machinery targeting too precise a cause is aggregated up to the parent causes; 2) a “bridge map” is performed over a specific set of sources to ensure that they do not contain causes that could not be reliably determined by the methods used.

Step 6: Drop VR country years or mark them as non-representative

Lozano and colleagues describe the negative impact of low-completeness VR data on CoD modelling for GBD 2010[4]. For GBD 2021, VR location-years with completeness less than 50% were dropped, and completeness between 50-69% was marked as non-representative. Moreover, any country-year with multiple mortalities registered to major garbage codes greater than 50% of the registered mortality was dropped.

Step 7: Aggregate causes

In this step, the cause list was organised into four levels in a top-down hierarchical format. For example, deaths are classified into three broad groups (Level 1 causes) that include “communicable, maternal, neonatal, nutritional diseases”, “non-communicable diseases”, and “injuries”. Within the Level 1 group of non-communicable diseases is the level 2 cause “diabetes and kidney diseases,” which aggregates the level 3 causes “Diabetes mellitus” and “chronic kidney disease”. “Diabetes mellitus” further aggregates the level 4 causes “Diabetes mellitus type 1” and “Diabetes mellitus type 2”; whilst “chronic kidney disease” aggregates give level 4 causes of “chronic kidney disease due to diabetes type 1”, “chronic kidney disease due to diabetes type 2”, “Hypertensive chronic kidney disease”, “glomerulonephritis chronic kidney disease” and “other chronic kidney disease”. This example demonstrates that the mortality estimate for a parent cause in the hierarchy represents the sum of the deaths due to the causes under that rubric. The parent Level 3 cause estimate includes deaths mapped directly to the parent and any Level 4 sub-causes.

Step 8: Application of noise reduction algorithm

Bayesian noise reduction algorithm was used to deal with zero counts in VR and VA for a specific age group in a specific year. Here, we assume normal prior and a normal data likelihood. Estimating normal prior for the given list of countries was done by running a Poisson regression to estimate the number of deaths due to a specific cause and sex with dummy variables for year and age.

Step 9: Identify outliers in the cause of death

As death rates for CoD tend to have a stable age pattern that does not change rapidly over time in a large population, it is fair to assume a relatively stable pattern in death rate for all causes. Rare exceptions to this include epidemic diseases and specific types of injuries. These outliers have been corrected using the noise reduction process as mentioned in step 8. Identifying outliers occurs before the models’ finalisation for each cause. This is based on the judgment of the modeller and senior faculty, and these outlier decisions are reversible and may be revisited.

4. Causes of Death Modelling Methods

To estimate mortality causes, GBD uses the Cause of Death Ensemble model (CODEm), which combines results from different statistical models weighted based on the out-of-sample predictive validity. The CODEm relies on four key components: First, all available data were identified and gathered to be used in the modelling process. Although these available data may differ in quality, all these data contained some signal of the actual epidemiological process. Second, diverse plausible models are implemented to obtain well-documented associations in the estimates. Using a wide range of individual models to create a predictive ensemble model has outperformed techniques using only a single model both in CoD estimation and in more general prediction applications. Third, the out-of-sample predictive validity is assessed for all individual models, which are then ranked for use in the ensemble modelling stage. Fourth, differently weighted combinations of individual models are examined to select the ensemble model with the highest out-of-sample predictive validity.

A range of plausible statistical models was developed for each cause as several factors may co-vary with any of the CoD. In the CODEm framework, four groups of statistical models are utilized: 1) linear mixed effects regression (LMER) models of the natural log of the cause-specific death rate, 2) LMER models of the logit of the cause fraction, 3) spatiotemporal Gaussian process regression (ST-GPR) models of the natural logarithm of the cause-specific death rate, and 4) SR-GPR models of the logit of the cause fraction. The component models are weighted according to their predictive validity rank to determine their contribution to the ensemble estimate. A set of ensemble models is then determined using the weights.

After the weighting scheme is decided, 1000 draws are created for the final ensemble, with the number of draws contributed by each model proportional to its weight. Mortality estimates were scaled with other causes of death to 100% of all-cause death estimates within each age, sex, year, and location. The estimate for each mortality cause is the mean of 1000 draws from the set best-performing models. 95% uncertainty intervals (UIs) were calculated for all estimates to reflect the 25th and 95th percentile values of the 1000 draws. DALYs were estimated by the summation of years of life lost and years lost due to disability, which serves to quantify the extent of health loss related to specific diseases. Years of life lost were obtained by multiplying the estimated number of deaths by age with a standard life expectancy. In contrast, the multiplication of prevalence computed years lost due to disability by a disability weight, ranging from 0 to 1 where 0 is a state of full health, and 1 is death. Age-standardized rates per 100,000 population were also derived using the direct method to the GBD 2021 population estimate with five-year age groups. All estimates were reported with the corresponding 95% UIs.

Supplementary Material S2: Sociodemographic index of national and subnational based on the GBD 2021 study.
(Countries are listed in **bold**, while subnational areas are listed in regular text.)

Low SDI

Addis Ababa. **Afghanistan**. Afar. Amhara. Bauchi. **Benin**. Benishangul-Gumuz. Bihar, Rural. Borno. **Burkina Faso**. **Burundi**. Balochistan. **Côte d'Ivoire**. **Central African Republic**. **Chad**. **Democratic Republic of the Congo**. Dire Dawa. **Eritrea**. **Gambia**. Gambella. Gilgit-Baltistan. Gombe. **Guinea**. **Guinea-Bissau**. **Haiti**. Harari. Jharkhand, Rural. Jigawa. Kaduna. Kano. Katsina. Kebbi. Khyber Pakhtunkhwa. **Liberia**. **Madagascar**. **Malawi**. **Mali**. Madhya Pradesh, Rural. **Mozambique**. **Nepal**. **Niger**. Oromia. **Papua New Guinea**. **Rwanda**. **Senegal**. **Sierra Leone**. Sokoto. **Solomon Islands**. **Somalia**. Southern Nations, Nationalities, and Peoples. **South Sudan**. Taraba. **Timor-Leste**. Tigray. **Togo**. **Uganda**. **United Republic of Tanzania**. **Yemen**. Yobe. Zamfara.

Low Middle SDI

Acre. Adamawa. Akwa Ibom. Alagoas. Amazonas. Andhra Pradesh, Rural. **Angola**. Arunachal Pradesh, Rural. Assam, Rural. Azad Jammu & Kashmir. Bahia. **Bangladesh**. Baringo. Bayelsa. **Belize**. Bengkulu. Benue. **Bhutan**. Bihar, Urban. **Bolivia (Plurinational State of)**. Bomet. Bungoma. Busia. **Cabo Verde**. **Cambodia**. **Cameroon**. Ceará. Central Java. Central Sulawesi. Chhattisgarh, Rural. **Comoros**. **Congo**. Cross River. Delta. **Democratic People's Republic of Korea**. **Djibouti**. East Nusa Tenggara. Ebonyi. **Egypt**. Ekiti. **El Salvador**. Elgeyo-Marakwet. Embu. Enugu. **Eswatini**. Garissa. **Ghana**. Gorontalo. Guatemala. Gujarat, Rural. Gujarat, Rural. Haryana, Rural. Homa Bay. **Honduras**. Isiolo. Jammu & Kashmir and Ladakh, Rural. Kajiado. Kakamega. Karnataka, Rural. Kericho. Kiambu. Kilifi. **Kiribati**. Kirinyaga. Kisii. Kisumu. Kitui. Kogi. Kwale. Kwara. **Kyrgyzstan**. Laikipia. Lampung. Lamu. **Lao People's Democratic Republic**. **Lesotho**. Machakos. Maharashtra, Rural. Makueni. Maluku. Mandera. Manipur, Rural. Maranhão. Marsabit. **Marshall Islands**. **Mauritania**. Meghalaya, Rural. Meru. **Micronesia (Federated States of)**. Migori. Mizoram, Rural. Mombasa. **Mongolia**. **Morocco**. Murang'a. **Myanmar**. Nagaland, Rural. Nairobi. Nakuru. **Namibia**. Nandi. Narok. Nasarawa. **Nicaragua**. North Maluku. Nyamira. Nyandarua. Nyeri. Odisha, Rural. Ogun. Ondo. Other Union Territories, Rural. Oyo. Paraíba. Pará. Pernambuco. Piauí. Plateau. Punjab. Punjab, Rural. Rajasthan, Rural. Rio Grande do Norte. Rondônia. Roraima. Samburu. **Samoa**. **Sao Tome and Principe**. Sergipe. Siaya. Sikkim, Rural. Sindh. Southeast Sulawesi. **Sudan**. Taita Taveta. **Tajikistan**. Tamil Nadu, Rural. Tana River. Telangana, Rural. Tharaka Nithi. Tibet. Tocantins. Trans Nzoia. Tripura, Rural. Turkana. **Tuvalu**. Uasin Gishu. Uttarakhand, Rural. Uttar Pradesh, Rural. **Vanuatu**. **Venezuela (Bolivarian Republic of**). Vihiga. Wajir. West Bengal, Rural. West Kalimantan. West Nusa Tenggara. West Pokot. West Sulawesi. **Zambia**. **Zimbabwe**.

Middle SDI

Abia. Abra. Aceh. Aguascalientes. Agusan Del Norte. Agusan Del Sur. Aklan. **Albania**. Albay. Alborz. **Algeria**. Amapá. Anambra. Andhra Pradesh, Urban. Anhui. Antique. Apayao. Ardebil. **Armenia**. Arunachal Pradesh, Urban. Assam, Urban. **Aurora**. **Azerbaijan**. Baja California. Baja California Sur. Bali. Bangka-Belitung Islands. Banten. Basilan. Bataan. Batanes. Batangas. Benguet. Biliran. Bohol. **Botswana**. Bukidnon. Bulacan. Bushehr. Cagayan. Camarines Norte. Camarines Sur. Camiguin. Campeche. Capiz. Catanduanes. Cavite. Cebu. Central Kalimantan. Chahar Mahaal and Bakhtiari. Chhattisgarh, Urban. Chiapas. Chihuahua. Coahuila. Colima. **Colombia**. **Costa Rica**. Cotabato (North Cotabato). **Cuba**. Davao de Oro. Davao Del Norte. Davao Del Sur. Davao Occidental. Davao Oriental. Delhi, Rural. Dinagat Islands. **Dominican Republic**. Durango. East Azarbayejan. East Java. Eastern Cape. Eastern Samar. **Ecuador**. Edo. **Equatorial Guinea**. Espírito Santo. Fars. FCT (Abuja). **Fiji**. Free State. **Gabon**. Gansu. Gauteng. Gilan. Goa, Rural. Goiás. Golestan. **Grenada**. Guanajuato. Guangxi. Guerrero. Guimaras. Guizhou. Gujarat, Urban. **Guyana**. Hainan. Hamadan. Haryana, Urban. Hebei. Henan. Hidalgo. Himachal Pradesh, Rural. Hormozgan. Hubei. Hunan. Ifugao. Ilam. Ilocos Norte. Ilocos Sur. Iloilo. Imo. **Iraq**. Isabela. Isfahan. Islamabad Capital Territory. Jalisco. **Jamaica**. Jambi. Jammu & Kashmir and Ladakh, Urban. Jharkhand, Urban. Jiangxi. Kalinga. Karnataka, Urban. Kerala. Kerala, Rural. Kerala, Urban. Kerman. Kermanshah. Khorasan-e-Razavi. Khuzestan. Kohgiluyeh and Boyer-Ahmad. Kurdistan. KwaZulu-Natal. La Union. Lagos. Laguna. Lanao Del Norte. Lanao Del Sur. Leyte. Limpopo. Lorestan. Madhya Pradesh, Urban. Maguindanao. Maharashtra, Urban. **Maldives**. Manipur, Urban. Marinduque. Markazi. Masbate. Mato Grosso. Mato Grosso do Sul. Mazandaran. Meghalaya, Urban. Mexico City. Michoacán. Michoacán de Ocampo. Minas Gerais. Misamis Occidental. Misamis Oriental. Mizoram, Urban. Morelos. Mountain Province. Mpumalanga. **México**. Nagaland, Urban. National Capital Region. **Nauru**. Nayarit. Negros Occidental. Negros Oriental. Ningxia. North Khorasan. North Sulawesi. North Sumatra. North-West. Northern Cape. Northern Samar. Nueva Ecija. Nueva Vizcaya. Nuevo León. Oaxaca. Occidental Mindoro. Odisha, Urban. Oriental Mindoro. Osun. Other Union Territories, Urban. Palawan. **Palestine**. Pampanga. **Panama**. Pangasinan. Papua. **Paraguay**. Paraná. **Peru**. Puebla. Punjab, Urban. Qazvin. Qinghai. Qom. Querétaro. Quezon. Quintana Roo. Quirino. Rajasthan, Urban. Rio de Janeiro. Rio Grande do Sul. Rivers. Rizal. Romblon. **Saint Lucia. Saint Vincent and the Grenadines**. Samar (Western Samar). San Luis Potosí. Santa Catarina. Sarangani. Semnan. Shanxi. Sichuan. Sikkim. Sikkim, Urban. Sinaloa. Siquijor. Sistan and Baluchistan. Sonora. Sorsogon. South Cotabato. South Kalimantan. South Khorasan. South Sulawesi. South Sumatra. Southern Leyte. **Sri Lanka**. Sultan Kudarat. Sulu. Surigao Del Norte. Surigao Del Sur. **Suriname**. **Syrian Arab Republic**. São Paulo. Tabasco. Tamaulipas. Tamil Nadu. Tamil Nadu, Urban. Tarlac. Tawi-Tawi. Tehran. Telangana. Telangana, Urban. **Thailand**. Tlaxcala. Tokelau. **Tonga**. Tripura. Tripura, Urban. **Tunisia**. **Turkmenistan**. Uttar Pradesh. Uttar Pradesh, Urban. **Uzbekistan**. Veracruz de Ignacio de la Llave. **Viet Nam**. West Azarbayejan. West Bengal. West Bengal, Urban. West Java. West Papua. West Sumatra. Western Cape. Xinjiang. Yazd. Yogyakarta. Yucatán. Yunnan. Zacatecas. Zambales. Zamboanga Del Norte. Zamboanga Del Sur. Zamboanga Sibugay. Zanjan.

High Middle SDI

Abruzzo. Altai Krai. American Samoa. Amur Oblast. **Antigua and Barbuda**. **Argentina**. Arkhangelsk oblast. Arkhangelsk oblast without Nenets autonomous district. Astrakhan Oblast. **Bahamas**. **Bahrain**. **Barbados**. Basilicata. **Belarus**. Belgorod Oblast. **Bosnia and Herzegovina**. **Brunei Darussalam**. Bryansk Oblast. **Bulgaria**. Calabria. Campania. Chechen Republic. Chelyabinsk oblast. **Chile**. Chongqing. Chukotka Autonomous Area. Chukotka Autonomous Okrug. Chuvash Republic. **Cook Islands**. **Croatia**. Delhi, Urban. Distrito Federal. **Dominica**. East Kalimantan. Emilia-Romagna. Friuli-Venezia Giulia. Fujian. **Georgia**. Goa, urban. **Greece**. **Guam**. Guangdong. Heilongjiang. Himachal Pradesh, urban. **Hungary**. Inner Mongolia. Irkutsk Oblast. **Israel**. Ivanovo Oblast. Jakarta. Jewish Autonomous Oblast. Jiangsu. Jilin. **Jordan**. Kabardino-Balkar Republic. Kaliningrad Oblast. Kaluga Oblast. Kamchatka Krai. Karachay-Cherkess Republic. **Kazakhstan**. Kemerovo Oblast. Khabarovsk Krai. Khanty-Mansi autonomous area. Kirov Oblast. Komi Republic. Kostroma Oblast. Krasnodar Krai. Krasnoyarsk Krai. Kurgan Oblast. Kursk Oblast. Lazio. **Lebanon**. Leningrad Oblast. Liaoning. **Libya**. Liguria. Lipetsk Oblast. Lipetzk oblast. Lombardia. Magadan Oblast. **Malaysia**. **Malta**. Marche. **Mauritius**. Molise. **Montenegro**. Moscow City. Moscow Oblast. Murmansk Oblast. Nenets autonomous district. **Niue**. Nizhny Novgorod Oblast. North Kalimantan. **North Macedonia**. Northern Mariana Islands. Novgorod Oblast. Novosibirsk Oblast. **Oman**. Omsk Oblast. Orenburg Oblast. Oryol Oblast. **Palau**. Penza Oblast. Perm Krai. Piemonte. **Portugal**. Primorsky Krai. Provincia autonoma di Bolzano. Provincia autonoma di Trento. Pskov Oblast. Puglia. Republic of Adygeya. Republic of Altai. Republic of Bashkortostan. Republic of Buryatia. Republic of Crimea. Republic of Dagestan. Republic of Ingushetia. Republic of Kalmykia. Republic of Karelia. Republic of Khakassia. Republic of Mari El. **Republic of Moldova**. Republic of Mordovia. Republic of North Ossetia-Alania. Republic of Sakha (Yakutia). Republic of Tatarstan. Republic of Tuva. Riau. Riau Islands. **Romania**. Rostov Oblast. Ryazan Oblast. **Saint Kitts and Nevis**. Saint Petersburg. Sakhalin Oblast. Samara Oblast. Saratov Oblast. Sardegna. **Serbia**. Sevastopol. **Seychelles**. Shaanxi. Shandong. Sicilia. **Slovakia**. Smolensk Oblast. **Spain**. Stavropol Krai. Sverdlovsk Oblast. Tambov Oblast. Tomsk Oblast. Toscana. **Trinidad and Tobago** Tula Oblast. Tver Oblast. Tyumen Oblast. Tyumen oblast without autonomous areas. **Türkiye**. Udmurt Republic. **Ukraine**. **Ukraine (without Crimea & Sevastopol)**. Ulyanovsk Oblast. Umbria. **Uruguay**. Uttarakhand. Uttarakhand, Urban. Valle d'Aosta. Veneto. Vladimir Oblast. Volgograd Oblast. Vologda Oblast. Voronezh Oblast. Yamalo-Nenets autonomous area. Yamalo-Nenets Autonomous Okrug. Yaroslavl Oblast. Zabaikalsk kray. Zhejiang.

High SDI

Agder. Aichi. Akita. Alabama. Alaska. **Andorra**. Aomori. Arizona. Arkansas. **Australia**. **Austria**. Barking and Dagenham. Barnet. Barnsley. Bath and North East Somerset. Bedford. Beijing. **Belgium**. Bermuda. Bexley. Birmingham. Blackburn with Darwen. Blackpool. Bolton. Bournemouth. Bracknell Forest. Bradford. Brent. Brighton and Hove. Bristol, City of. Bromley. Buckinghamshire. Bury. Calderdale. California. Cambridgeshire. Camden. **Canada**. Central Bedfordshire. Cheshire East. Cheshire West and Chester. Chiba. Colorado. Connecticut. Cornwall. County Durham. Coventry. Croydon. Cumbria. **Cyprus**. **Czechia**. Darlington. Delaware. **Denmark**. Derby. Derbyshire. Devon. District of Columbia. Dolnośląskie. Doncaster. Dorset. Dudley. Ealing. East Riding of Yorkshire. East Sussex. Ehime. Enfield. Essex. **Estonia**. **Finland**. Florida. **France**. Fukui. Fukuoka. Fukushima. Gateshead. **Georgia. Germany**. Gifu. Gloucestershire. **Greenland**. Greenwich. Gunma. Hackney. Halton. Hammersmith and Fulham. Hampshire. Haringey. Harrow. Hartlepool. Havering. Hawaii. Herefordshire, County of. Hertfordshire. Hillingdon. Hiroshima. Hokkaidō. Hong Kong. Hong Kong Special Administrative Region of China. Hounslow. Hyōgo. Ibaraki. **Iceland**. Idaho. Illinois. Indiana. Innlandet. Iowa. **Ireland**. Ishikawa. Isle of Wight.

Islington. Iwate. Kagawa. Kagoshima. Kanagawa. Kansas. Kensington and Chelsea. Kent. Kentucky. Kingston upon Hull, City of. Kingston upon Thames. Kirklees. Knowsley. Kujawsko-Pomorskie. Kumamoto. **Kuwait**. Kyoto. Kōchi. Lambeth. Lancashire. **Latvia**. Leeds. Leicester. Leicestershire. Lewisham. Lincolnshire. **Lithuania**. Liverpool. Łódzkie. Louisiana. Lubelskie. Lubuskie. Luton. **Luxembourg**. Macao. Macao Special Administrative Region of China. Maine. Manchester. Maryland. Massachusetts. Mazowieckie. Małopolskie. Medway. Merton. Michigan. Middlesbrough. Mie. Milton Keynes. Minnesota. Mississippi. Missouri. Miyagi. Miyazaki. **Monaco**. Montana. Møre og Romsda. Nagano. Nagasaki. Nara. Nebraska. **Netherlands**. Nevada. New Hampshire. New Jersey. New Mexico. New York. **New Zealand**. New Zealand Maori population. New Zealand non-Maori population. Newcastle upon Tyne. Newham. Niigata. Nordland. Norfolk. North Carolina. North Dakota. North East Lincolnshire. North Lincolnshire. North Somerset. North Tyneside. North Yorkshire. Northamptonshire. **Northern Ireland**. Northumberland. Nottingham. Nottinghamshire. Ohio. Oita. Okayama. Okinawa. Oklahoma. Oldham. Opolskie. Oregon. Osaka. Oslo. Oxfordshire. Pennsylvania. Peterborough. Plymouth. Podkarpackie. Podlaskie. Pomorskie. Poole. Portsmouth. Puerto Rico. **Qatar**. Reading. Redbridge. Redcar and Cleveland. **Republic of Korea**. Rhode Island. Richmond upon Thames. Rochdale. Rogaland. Rotherham. Rutland. Saga. Saitama. Salford. **San Marino**. Sandwell. **Saudi Arabia**. **Scotland**. Sefton. Shanghai. Sheffield. Shiga. Shimane. Shizuoka. Shropshire. **Singapore**. Śląskie. Slough. **Slovenia**. Solihul. Somerset. South Carolina. South Dakota. South Gloucestershire. South Tyneside. Southampton. Southend-on-Sea. Southwark. St Helens. Staffordshire. Stockholm. Stockport. Stockton-on-Tees. Stoke-on-Trent. Suffolk. Sunderland. Surrey. Sutton. **Sweden**. Sweden except Stockholm. Świętokrzyskie. Swindon. **Switzerland**. **Taiwan**. Tameside. Telford and Wrekin. Tennessee. Texas. Thurrock. Tianjin. Tochigi. Tokushima. Tokyo. Torbay. Tottori. Tower Hamlets. Toyama. Trafford. Troms og Finnmark. Trøndelag. **United Arab Emirates**. United States Virgin Islands. Utah. Vermont. Vestfold og Telemark. Vestland. Viken. Virginia. Wakayama. Wakefield. **Wales**. Walsall. Waltham Forest. Wandsworth. Warmińsko-Mazurskie. Warrington. Warwickshire. Washington. West Berkshire. West Sussex. West Virginia. Westminster. Wielkopolskie. Wigan. Wiltshire. Windsor and Maidenhead. Wirral. Wisconsin. Wokingham. Wolverhampton. Worcestershire. Wyoming. Yamagata. Yamaguchi. Yamanashi. York. Zachodniopomorskie.

The subnational sociodemographic Index can be accessed at https://doi.org/10.1016/S0140-6736(24)00757-8

Supplementary Table S1 Incidence and age-standardized rates of patients with alcohol associated primary liver cancer in young adults in 2000 and 2021 and changes from 2000 to 2021

Country	2000 Incidence (95% UI)	2000 Age-standardized incidence rate (95% UI)	2021 Incidence (95% UI)	2021 Age-standardized incidence rate (95% UI)	2000 to 2021 Annual Percent Change (95% CI)	"P-Value"
American Samoa	0 (0 to 0)	0.09 (0.05 to 0.17)	0.04 (0.02 to 0.07)	0.15 (0.07 to 0.28)	2.33 (2.2 to 2.45)	<0.001
Antigua and Barbuda	0 (0 to 0)	0.09 (0.05 to 0.14)	0.05 (0.03 to 0.08)	0.11 (0.07 to 0.16)	0.93 (0.77 to 1.08)	<0.001
Arab Republic of Egypt	50 (20 to 90)	0.14 (0.07 to 0.25)	87.35 (46.74 to 152.1)	0.16 (0.09 to 0.28)	0.75 (0.59 to 0.92)	<0.001
Argentine Republic	10 (0 to 10)	0.03 (0.02 to 0.05)	12.29 (7.62 to 19.9)	0.05 (0.03 to 0.08)	2.06 (1.87 to 2.24)	<0.001
Australia	30 (20 to 40)	0.3 (0.2 to 0.4)	60.18 (39.98 to 84.9)	0.5 (0.33 to 0.71)	2.54 (2.47 to 2.62)	<0.001
Barbados	0 (0 to 0)	0.12 (0.07 to 0.18)	0.21 (0.12 to 0.33)	0.15 (0.08 to 0.24)	0.88 (0.74 to 1.02)	<0.001
Belize	0 (0 to 0)	0.09 (0.05 to 0.13)	0.26 (0.16 to 0.39)	0.11 (0.07 to 0.16)	1.01 (0.65 to 1.38)	<0.001
Bermuda	0 (0 to 0)	0.12 (0.08 to 0.18)	0.03 (0.02 to 0.05)	0.13 (0.07 to 0.19)	0.05 (-0.16 to 0.26)	0.662
Bolivarian Republic of Venezuela	10 (10 to 10)	0.08 (0.05 to 0.11)	18.32 (10.68 to 28.52)	0.14 (0.08 to 0.22)	2.93 (2.68 to 3.18)	<0.001
Bosnia and Herzegovina	10 (0 to 10)	0.28 (0.15 to 0.45)	3.32 (1.78 to 5.64)	0.22 (0.12 to 0.38)	-1.06 (-1.36 to -0.76)	<0.001
Brunei Darussalam	0 (0 to 0)	0.1 (0.04 to 0.18)	0.32 (0.14 to 0.64)	0.12 (0.05 to 0.23)	0.84 (0.75 to 0.93)	<0.001
Burkina Faso	40 (10 to 90)	0.83 (0.28 to 1.78)	73.07 (26.39 to 149.63)	0.71 (0.26 to 1.45)	-0.78 (-1.06 to -0.49)	<0.001
Canada	60 (50 to 80)	0.41 (0.31 to 0.53)	80.85 (58.97 to 104.3)	0.49 (0.35 to 0.63)	0.78 (0.7 to 0.86)	<0.001
Central African Republic	10 (0 to 10)	0.32 (0.11 to 0.72)	5.01 (1.69 to 10.73)	0.19 (0.06 to 0.4)	-2.47 (-2.67 to -2.26)	<0.001
Commonwealth of Dominica	0 (0 to 0)	0.13 (0.08 to 0.21)	0.05 (0.02 to 0.08)	0.14 (0.07 to 0.24)	0.35 (0.25 to 0.46)	<0.001
Commonwealth of the Bahamas	0 (0 to 0)	0.19 (0.12 to 0.29)	0.44 (0.26 to 0.72)	0.21 (0.12 to 0.34)	0.48 (0.26 to 0.69)	<0.001
Cook Islands	0 (0 to 0)	0.23 (0.11 to 0.45)	0.05 (0.02 to 0.1)	0.63 (0.3 to 1.18)	4.85 (4.72 to 4.98)	<0.001
Czech Republic	10 (10 to 20)	0.26 (0.18 to 0.36)	8.01 (5.03 to 12.05)	0.17 (0.11 to 0.26)	-2.06 (-2.58 to -1.53)	<0.001
Democratic People's Republic of Korea	30 (10 to 50)	0.21 (0.09 to 0.43)	25.9 (11.68 to 51.6)	0.19 (0.08 to 0.37)	-0.53 (-0.64 to -0.43)	<0.001
Democratic Republic of Sao Tome and Principe	0 (0 to 0)	0.1 (0.05 to 0.17)	0.1 (0.05 to 0.23)	0.09 (0.04 to 0.2)	-0.31 (-0.57 to -0.05)	0.021
Democratic Republic of the Congo	20 (10 to 40)	0.07 (0.02 to 0.17)	27.7 (10 to 70.92)	0.06 (0.02 to 0.16)	-0.45 (-0.56 to -0.34)	<0.001
Democratic Republic of Timor-Leste	0 (0 to 0)	0.07 (0.03 to 0.13)	0.47 (0.21 to 1.04)	0.07 (0.03 to 0.15)	0.21 (0.11 to 0.31)	<0.001
Democratic Socialist Republic of Sri Lanka	10 (0 to 10)	0.07 (0.04 to 0.12)	6.7 (3.18 to 12.64)	0.06 (0.03 to 0.11)	-0.98 (-1.39 to -0.58)	<0.001
Dominican Republic	0 (0 to 10)	0.07 (0.04 to 0.12)	6.71 (3.51 to 11.23)	0.11 (0.06 to 0.19)	2.15 (2.01 to 2.3)	<0.001
Eastern Republic of Uruguay	0 (0 to 0)	0.04 (0.02 to 0.06)	1.67 (0.94 to 2.71)	0.1 (0.06 to 0.17)	4.64 (4.31 to 4.96)	<0.001
Federal Democratic Republic of Ethiopia	20 (10 to 30)	0.06 (0.04 to 0.1)	28.26 (17.34 to 46.38)	0.05 (0.03 to 0.08)	-1.11 (-1.44 to -0.78)	<0.001
Federal Democratic Republic of Nepal	10 (0 to 10)	0.06 (0.03 to 0.11)	19.67 (10.17 to 34.69)	0.12 (0.06 to 0.21)	3.2 (3.05 to 3.36)	<0.001
Federal Republic of Germany	120 (90 to 160)	0.3 (0.21 to 0.41)	102.07 (66.45 to 145.51)	0.29 (0.19 to 0.41)	-0.3 (-0.41 to -0.19)	<0.001
Federal Republic of Nigeria	70 (30 to 140)	0.13 (0.05 to 0.24)	101.9 (60.53 to 169.07)	0.09 (0.06 to 0.16)	-1.48 (-1.61 to -1.36)	<0.001
Federal Republic of Somalia	10 (0 to 20)	0.23 (0.07 to 0.52)	17.73 (4.99 to 43.75)	0.18 (0.05 to 0.44)	-1.33 (-1.64 to -1.03)	<0.001
Federated States of Micronesia	0 (0 to 0)	0.14 (0.06 to 0.3)	0.07 (0.03 to 0.16)	0.13 (0.06 to 0.29)	-0.22 (-0.48 to 0.04)	0.101
Federative Republic of Brazil	80 (70 to 100)	0.09 (0.07 to 0.11)	103.18 (79.89 to 126.88)	0.09 (0.07 to 0.11)	0.07 (-0.1 to 0.23)	0.43
French Republic	110 (70 to 160)	0.38 (0.24 to 0.54)	94.61 (57.72 to 145.62)	0.33 (0.2 to 0.51)	-0.56 (-0.7 to -0.41)	<0.001

Gabonese Republic	0 (0 to 10)	0.38 (0.16 to 0.86)	3.02 (1.23 to 5.84)	0.33 (0.13 to 0.63)	-0.78 (-0.93 to -0.62)	<0.001
Georgia	0 (0 to 10)	0.18 (0.11 to 0.28)	3.82 (2.28 to 5.97)	0.24 (0.14 to 0.37)	1.28 (1.08 to 1.48)	<0.001
Grand Duchy of Luxembourg	0 (0 to 0)	0.24 (0.16 to 0.34)	0.64 (0.39 to 0.91)	0.2 (0.12 to 0.29)	-0.87 (-1.18 to -0.55)	<0.001
Greenland	0 (0 to 0)	0.47 (0.29 to 0.71)	0.1 (0.05 to 0.16)	0.36 (0.18 to 0.61)	-1.27 (-1.4 to -1.14)	<0.001
Grenada	0 (0 to 0)	0.11 (0.06 to 0.16)	0.1 (0.06 to 0.16)	0.2 (0.12 to 0.3)	2.85 (2.73 to 2.97)	<0.001
Guam	0 (0 to 0)	0.11 (0.06 to 0.19)	0.15 (0.08 to 0.26)	0.21 (0.11 to 0.35)	2.93 (2.78 to 3.08)	<0.001
Hashemite Kingdom of Jordan	0 (0 to 0)	0.01 (0.01 to 0.02)	0.88 (0.44 to 1.67)	0.01 (0.01 to 0.02)	0.06 (-0.16 to 0.27)	0.616
Hellenic Republic	10 (0 to 10)	0.14 (0.09 to 0.22)	10.54 (6.18 to 16.62)	0.24 (0.14 to 0.38)	2.63 (2.5 to 2.75)	<0.001
Hungary	10 (10 to 20)	0.23 (0.14 to 0.35)	5.02 (2.95 to 7.66)	0.12 (0.07 to 0.18)	-3.26 (-3.47 to -3.04)	<0.001
Independent State of Papua New Guinea	0 (0 to 0)	0.05 (0.02 to 0.16)	2.48 (0.85 to 6.83)	0.05 (0.02 to 0.13)	-0.8 (-0.82 to -0.78)	<0.001
Independent State of Samoa	0 (0 to 0)	0.08 (0.04 to 0.17)	0.09 (0.04 to 0.18)	0.09 (0.04 to 0.18)	0.13 (-0.02 to 0.27)	0.083
Ireland	0 (0 to 0)	0.15 (0.1 to 0.21)	4.06 (2.62 to 5.9)	0.18 (0.11 to 0.26)	0.79 (0.64 to 0.95)	<0.001
Islamic Republic of Afghanistan	0 (0 to 0)	0.04 (0.02 to 0.08)	7.53 (3.62 to 15.13)	0.05 (0.02 to 0.1)	0.96 (0.63 to 1.29)	<0.001
Islamic Republic of Iran	0 (0 to 10)	0.01 (0.01 to 0.02)	10.82 (7.55 to 15.03)	0.02 (0.02 to 0.03)	2.86 (2.15 to 3.57)	<0.001
Islamic Republic of Mauritania	10 (0 to 20)	0.69 (0.14 to 1.73)	7.77 (2.88 to 15.69)	0.38 (0.14 to 0.76)	-2.85 (-3.01 to -2.69)	<0.001
Islamic Republic of Pakistan	80 (50 to 110)	0.12 (0.08 to 0.17)	148.2 (100.3 to 211.07)	0.12 (0.08 to 0.17)	0.1 (0.04 to 0.15)	<0.001
Jamaica	0 (0 to 0)	0.05 (0.03 to 0.08)	1.07 (0.56 to 1.83)	0.07 (0.04 to 0.12)	1.09 (0.81 to 1.38)	<0.001
Japan	260 (200 to 330)	0.43 (0.33 to 0.54)	90.03 (66.96 to 119.64)	0.18 (0.13 to 0.24)	-4.09 (-4.28 to -3.89)	<0.001
Kingdom of Bahrain	0 (0 to 0)	0.05 (0.02 to 0.08)	0.36 (0.17 to 0.67)	0.04 (0.02 to 0.07)	-1.49 (-2.09 to -0.88)	<0.001
Kingdom of Belgium	10 (10 to 20)	0.24 (0.16 to 0.33)	12.76 (8.22 to 18.51)	0.26 (0.16 to 0.37)	0.4 (0.28 to 0.53)	<0.001
Kingdom of Bhutan	0 (0 to 0)	0.13 (0.06 to 0.24)	0.62 (0.27 to 1.24)	0.14 (0.06 to 0.28)	0.55 (0.4 to 0.7)	<0.001
Kingdom of Cambodia	10 (0 to 30)	0.19 (0.08 to 0.47)	24.27 (9.47 to 53.83)	0.27 (0.1 to 0.6)	1.56 (1.47 to 1.66)	<0.001
Kingdom of Denmark	10 (0 to 10)	0.21 (0.13 to 0.29)	2.84 (1.83 to 4.16)	0.11 (0.07 to 0.16)	-2.91 (-3.26 to -2.57)	<0.001
Kingdom of Eswatini	0 (0 to 10)	0.89 (0.36 to 1.79)	6.21 (1.54 to 18.33)	1.01 (0.25 to 2.97)	0.58 (0.43 to 0.73)	<0.001
Kingdom of Lesotho	0 (0 to 10)	0.49 (0.18 to 1.21)	6.85 (1.68 to 20.33)	0.68 (0.17 to 2.02)	1.5 (1.36 to 1.64)	<0.001
Kingdom of Morocco	0 (0 to 0)	0 (0 to 0.01)	1.23 (0.54 to 2.54)	0.01 (0 to 0.01)	1.74 (1.69 to 1.78)	<0.001
Kingdom of Norway	0 (0 to 0)	0.15 (0.12 to 0.19)	7.29 (5.64 to 9.03)	0.29 (0.23 to 0.36)	3.07 (2.93 to 3.22)	<0.001
Kingdom of Saudi Arabia	0 (0 to 10)	0.03 (0.01 to 0.05)	5.4 (2.23 to 10.83)	0.02 (0.01 to 0.04)	-1.23 (-1.72 to -0.73)	<0.001
Kingdom of Spain	70 (50 to 110)	0.35 (0.23 to 0.49)	80.45 (47.39 to 126.22)	0.4 (0.24 to 0.63)	0.73 (0.56 to 0.9)	<0.001
Kingdom of Sweden	0 (0 to 0)	0.1 (0.08 to 0.12)	9.32 (6.9 to 12.1)	0.21 (0.15 to 0.27)	3.59 (3.26 to 3.93)	<0.001
Kingdom of Thailand	400 (220 to 630)	1.1 (0.6 to 1.74)	368.76 (194.8 to 628.78)	1.16 (0.61 to 1.97)	0.22 (0.03 to 0.4)	0.022
Kingdom of the Netherlands	10 (10 to 10)	0.13 (0.09 to 0.18)	10 (6.51 to 14.28)	0.14 (0.09 to 0.19)	0.23 (0.1 to 0.36)	<0.001
Kingdom of Tonga	0 (0 to 0)	0.53 (0.26 to 0.95)	0.24 (0.11 to 0.48)	0.5 (0.22 to 0.97)	-0.31 (-0.44 to -0.17)	<0.001
Kyrgyz Republic	0 (0 to 10)	0.12 (0.06 to 0.2)	2.86 (1.52 to 4.93)	0.08 (0.04 to 0.14)	-1.79 (-2.23 to -1.35)	<0.001
Lao People's Democratic Republic	10 (0 to 20)	0.33 (0.16 to 0.6)	12.47 (5.92 to 21.65)	0.31 (0.15 to 0.54)	-0.21 (-0.28 to -0.14)	<0.001
Lebanese Republic	0 (0 to 0)	0.02 (0.01 to 0.04)	0.74 (0.36 to 1.44)	0.02 (0.01 to 0.05)	0.7 (0.34 to 1.06)	<0.001

Malaysia	10 (10 to 20)	0.1 (0.05 to 0.17)	20.91 (9.7 to 35.81)	0.12 (0.05 to 0.2)	0.75 (0.66 to 0.84)	<0.001
Mongolia	20 (10 to 40)	1.6 (0.73 to 2.96)	46.31 (23.94 to 82.04)	2.75 (1.42 to 4.86)	2.73 (2.42 to 3.04)	<0.001
Montenegro	0 (0 to 0)	0.34 (0.2 to 0.51)	0.98 (0.56 to 1.56)	0.33 (0.19 to 0.53)	-0.05 (-0.22 to 0.13)	0.615
New Zealand	10 (10 to 10)	0.46 (0.35 to 0.57)	15.08 (11.42 to 19.03)	0.62 (0.47 to 0.78)	1.45 (1.2 to 1.7)	<0.001
North Macedonia	0 (0 to 10)	0.38 (0.23 to 0.6)	3.73 (2 to 6.24)	0.34 (0.18 to 0.57)	-0.64 (-0.8 to -0.48)	<0.001
Northern Mariana Islands	0 (0 to 0)	0.1 (0.05 to 0.17)	0.04 (0.01 to 0.07)	0.15 (0.06 to 0.3)	2.33 (1.74 to 2.92)	<0.001
Palestine	0 (0 to 0)	0.04 (0.02 to 0.07)	0.94 (0.5 to 1.68)	0.04 (0.02 to 0.06)	-0.28 (-0.41 to -0.14)	<0.001
People's Democratic Republic of Algeria	0 (0 to 0)	0.01 (0.01 to 0.02)	5.49 (2.63 to 11.19)	0.02 (0.01 to 0.05)	2.92 (2.8 to 3.04)	<0.001
People's Republic of Bangladesh	40 (20 to 70)	0.06 (0.03 to 0.11)	56.69 (26.44 to 109.42)	0.06 (0.03 to 0.12)	0.71 (0.67 to 0.75)	<0.001
People's Republic of China	2050 (1480 to 2710)	0.28 (0.21 to 0.37)	2237.8 (1472.5 to 3147.84)	0.34 (0.22 to 0.47)	0.88 (0.52 to 1.24)	<0.001
Plurinational State of Bolivia	0 (0 to 10)	0.07 (0.04 to 0.13)	5.2 (2.57 to 9.72)	0.08 (0.04 to 0.16)	0.81 (0.68 to 0.95)	<0.001
Portuguese Republic	10 (10 to 20)	0.26 (0.18 to 0.39)	18.52 (11.41 to 28.32)	0.4 (0.25 to 0.62)	2.02 (1.85 to 2.19)	<0.001
Principality of Andorra	0 (0 to 0)	0.71 (0.38 to 1.17)	0.33 (0.16 to 0.55)	0.79 (0.4 to 1.34)	0.52 (0.37 to 0.67)	<0.001
Principality of Monaco	0 (0 to 0)	0.46 (0.25 to 0.8)	0.09 (0.05 to 0.15)	0.63 (0.32 to 1.07)	1.47 (1.12 to 1.82)	<0.001
Puerto Rico	0 (0 to 0)	0.15 (0.09 to 0.22)	3.12 (1.78 to 5.04)	0.21 (0.12 to 0.34)	1.63 (1.38 to 1.89)	<0.001
Republic of Albania	0 (0 to 10)	0.29 (0.16 to 0.48)	3.93 (1.94 to 7)	0.31 (0.15 to 0.56)	0.38 (0.27 to 0.5)	<0.001
Republic of Angola	20 (0 to 60)	0.26 (0.03 to 0.85)	32.33 (5.82 to 85.67)	0.22 (0.04 to 0.58)	-0.73 (-0.82 to -0.64)	<0.001
Republic of Armenia	0 (0 to 10)	0.21 (0.12 to 0.35)	3.27 (1.9 to 5.17)	0.22 (0.13 to 0.35)	0.36 (0.16 to 0.56)	<0.001
Republic of Austria	20 (10 to 20)	0.44 (0.33 to 0.56)	20.32 (15.01 to 26.74)	0.5 (0.37 to 0.66)	0.66 (0.5 to 0.81)	<0.001
Republic of Azerbaijan	10 (10 to 20)	0.22 (0.12 to 0.37)	17.42 (7.54 to 35.71)	0.31 (0.14 to 0.64)	1.81 (1.69 to 1.93)	<0.001
Republic of Belarus	10 (10 to 20)	0.2 (0.12 to 0.29)	10.06 (5.75 to 16.02)	0.24 (0.14 to 0.38)	0.93 (0.69 to 1.17)	<0.001
Republic of Benin	20 (10 to 30)	0.56 (0.23 to 1.08)	20.78 (10.13 to 37.15)	0.33 (0.16 to 0.59)	-2.54 (-2.82 to -2.25)	<0.001
Republic of Botswana	0 (0 to 10)	0.31 (0.08 to 0.99)	3.41 (1.17 to 8.92)	0.25 (0.09 to 0.66)	-1.03 (-1.23 to -0.83)	<0.001
Republic of Bulgaria	20 (10 to 20)	0.41 (0.26 to 0.58)	12.09 (7.42 to 18.36)	0.41 (0.25 to 0.63)	0.1 (-0.13 to 0.33)	0.412
Republic of Burundi	0 (0 to 10)	0.16 (0.07 to 0.32)	4.65 (2 to 10.05)	0.07 (0.03 to 0.16)	-3.54 (-3.91 to -3.17)	<0.001
Republic of C√¥te d'Ivoire	20 (10 to 30)	0.18 (0.1 to 0.32)	17.22 (7.53 to 32.86)	0.13 (0.05 to 0.24)	-1.73 (-2.17 to -1.29)	<0.001
Republic of Cabo Verde	0 (0 to 0)	0.4 (0.2 to 0.7)	1.63 (0.81 to 2.95)	0.52 (0.26 to 0.94)	1.26 (1.17 to 1.34)	<0.001
Republic of Cameroon	40 (20 to 80)	0.57 (0.27 to 1.16)	69.1 (29.95 to 142.21)	0.45 (0.19 to 0.92)	-1.23 (-1.45 to -1.01)	<0.001
Republic of Chad	20 (10 to 40)	0.52 (0.2 to 1.11)	24.36 (11.29 to 50.15)	0.33 (0.15 to 0.68)	-2.2 (-2.39 to -2.02)	<0.001
Republic of Chile	0 (0 to 10)	0.05 (0.03 to 0.07)	9.01 (5.28 to 13.94)	0.09 (0.06 to 0.15)	3.34 (3.25 to 3.43)	<0.001
Republic of Colombia	10 (10 to 20)	0.05 (0.03 to 0.08)	16.85 (9.96 to 26.45)	0.06 (0.04 to 0.1)	0.84 (0.71 to 0.97)	<0.001
Republic of Costa Rica	0 (0 to 10)	0.18 (0.11 to 0.26)	5.32 (3.33 to 7.97)	0.21 (0.13 to 0.32)	0.86 (0.55 to 1.18)	<0.001
Republic of Croatia	0 (0 to 10)	0.21 (0.13 to 0.31)	2.44 (1.43 to 3.73)	0.13 (0.08 to 0.2)	-2.23 (-2.88 to -1.58)	<0.001
Republic of Cuba	10 (0 to 10)	0.09 (0.06 to 0.14)	5.15 (3.05 to 7.86)	0.1 (0.06 to 0.15)	0.27 (-0.06 to 0.59)	0.106
Republic of Cyprus	0 (0 to 0)	0.17 (0.1 to 0.25)	0.95 (0.53 to 1.57)	0.13 (0.08 to 0.22)	-1.12 (-1.23 to -1.02)	<0.001
Republic of Djibouti	0 (0 to 0)	0.13 (0.06 to 0.24)	0.91 (0.37 to 1.78)	0.13 (0.05 to 0.26)	0.15 (0.02 to 0.28)	0.022
Republic of Ecuador	0 (0 to 10)	0.05 (0.03 to 0.08)	6.55 (3.57 to 10.9)	0.07 (0.04 to 0.12)	1.55 (1.28 to 1.82)	<0.001

Republic of El Salvador	0 (0 to 0)	0.04 (0.03 to 0.06)	2.42 (1.38 to 3.82)	0.07 (0.04 to 0.12)	2.48 (2.03 to 2.93)	<0.001
Republic of Equatorial Guinea	0 (0 to 0)	0.06 (0.02 to 0.15)	1.01 (0.41 to 2.09)	0.12 (0.05 to 0.26)	3.8 (3.53 to 4.06)	<0.001
Republic of Estonia	0 (0 to 0)	0.29 (0.18 to 0.42)	1.39 (0.85 to 2)	0.24 (0.15 to 0.35)	-0.83 (-0.89 to -0.76)	<0.001
Republic of Fiji	0 (0 to 0)	0.06 (0.03 to 0.11)	0.5 (0.23 to 0.92)	0.11 (0.05 to 0.2)	2.66 (2.42 to 2.91)	<0.001
Republic of Finland	10 (0 to 10)	0.27 (0.17 to 0.39)	5.56 (3.53 to 8.34)	0.24 (0.15 to 0.36)	-0.53 (-0.77 to -0.29)	<0.001
Republic of Ghana	50 (20 to 80)	0.52 (0.27 to 0.92)	48.36 (22.1 to 91.97)	0.28 (0.13 to 0.52)	-2.93 (-3.25 to -2.6)	<0.001
Republic of Guatemala	0 (0 to 10)	0.09 (0.06 to 0.14)	13.15 (8.04 to 20.25)	0.16 (0.1 to 0.24)	2.41 (2.18 to 2.64)	<0.001
Republic of Guinea	20 (10 to 40)	0.61 (0.33 to 1.04)	27.39 (13.02 to 52.51)	0.45 (0.21 to 0.86)	-1.44 (-1.55 to -1.33)	<0.001
Republic of Guinea-Bissau	10 (0 to 10)	0.94 (0.29 to 1.9)	6.14 (2.92 to 11.54)	0.61 (0.29 to 1.15)	-2 (-2.11 to -1.89)	<0.001
Republic of Guyana	0 (0 to 0)	0.16 (0.1 to 0.23)	0.56 (0.31 to 0.92)	0.14 (0.08 to 0.23)	-0.79 (-1.35 to -0.23)	0.006
Republic of Haiti	0 (0 to 0)	0.05 (0.02 to 0.1)	3.18 (1.26 to 7.08)	0.05 (0.02 to 0.1)	-0.12 (-0.2 to -0.05)	0.002
Republic of Honduras	0 (0 to 0)	0.05 (0.03 to 0.1)	3.23 (1.56 to 6)	0.06 (0.03 to 0.11)	0.49 (0.37 to 0.6)	<0.001
Republic of Iceland	0 (0 to 0)	0.13 (0.08 to 0.19)	0.3 (0.19 to 0.45)	0.18 (0.11 to 0.27)	1.65 (1.48 to 1.81)	<0.001
Republic of India	480 (350 to 630)	0.09 (0.07 to 0.12)	950.8 (704.43 to 1266.07)	0.12 (0.09 to 0.16)	1.46 (1.41 to 1.51)	<0.001
Republic of Indonesia	160 (100 to 240)	0.13 (0.08 to 0.21)	252.73 (152.27 to 425.93)	0.16 (0.1 to 0.28)	0.98 (0.96 to 1.01)	<0.001
Republic of Iraq	0 (0 to 10)	0.03 (0.01 to 0.06)	9.16 (4.44 to 18.49)	0.04 (0.02 to 0.08)	1.34 (1.18 to 1.5)	<0.001
Republic of Italy	90 (70 to 110)	0.32 (0.26 to 0.39)	74.48 (57.75 to 93.87)	0.3 (0.23 to 0.38)	-0.35 (-0.79 to 0.1)	0.128
Republic of Kazakhstan	50 (30 to 80)	0.62 (0.38 to 0.95)	19.68 (11.92 to 30.51)	0.21 (0.13 to 0.33)	-5.19 (-5.76 to -4.61)	<0.001
Republic of Kenya	20 (10 to 30)	0.11 (0.07 to 0.19)	38.55 (25.31 to 58.69)	0.15 (0.1 to 0.22)	1.58 (1.43 to 1.72)	<0.001
Republic of Kiribati	0 (0 to 0)	0.15 (0.07 to 0.28)	0.07 (0.03 to 0.15)	0.11 (0.05 to 0.24)	-1.09 (-1.24 to -0.95)	<0.001
Republic of Korea	230 (130 to 380)	0.83 (0.47 to 1.4)	178.49 (89.61 to 323.4)	0.73 (0.37 to 1.33)	-0.59 (-0.86 to -0.33)	<0.001
Republic of Latvia	0 (0 to 0)	0.2 (0.12 to 0.3)	2.24 (1.39 to 3.44)	0.28 (0.18 to 0.44)	1.8 (1.52 to 2.08)	<0.001
Republic of Liberia	10 (0 to 20)	0.61 (0.24 to 1.28)	18.32 (8.38 to 35.28)	0.65 (0.3 to 1.26)	0.32 (0.22 to 0.43)	<0.001
Republic of Lithuania	0 (0 to 0)	0.18 (0.11 to 0.27)	3.48 (2.13 to 5.19)	0.3 (0.18 to 0.45)	2.48 (1.97 to 3)	<0.001
Republic of Madagascar	10 (0 to 10)	0.08 (0.04 to 0.17)	10.33 (4.54 to 20.94)	0.07 (0.03 to 0.15)	-0.66 (-0.75 to -0.56)	<0.001
Republic of Malawi	10 (0 to 20)	0.18 (0.09 to 0.35)	14.89 (7.17 to 28.48)	0.15 (0.07 to 0.29)	-0.86 (-0.96 to -0.77)	<0.001
Republic of Maldives	0 (0 to 0)	0.09 (0.04 to 0.16)	0.32 (0.16 to 0.57)	0.09 (0.05 to 0.17)	0.26 (-0.17 to 0.7)	0.238
Republic of Mali	30 (10 to 50)	0.56 (0.27 to 1.09)	56.39 (28.05 to 101.38)	0.54 (0.27 to 0.97)	-0.28 (-0.38 to -0.18)	<0.001
Republic of Malta	0 (0 to 0)	0.1 (0.06 to 0.16)	0.28 (0.17 to 0.43)	0.14 (0.09 to 0.22)	1.52 (1.33 to 1.71)	<0.001
Republic of Mauritius	0 (0 to 0)	0.05 (0.03 to 0.08)	0.19 (0.11 to 0.31)	0.03 (0.02 to 0.05)	-2.77 (-3.12 to -2.42)	<0.001
Republic of Moldova	10 (0 to 10)	0.29 (0.19 to 0.41)	4.62 (2.92 to 6.57)	0.26 (0.16 to 0.37)	-0.53 (-0.86 to -0.19)	0.002
Republic of Mozambique	30 (10 to 50)	0.36 (0.17 to 0.69)	54.81 (19.74 to 132.99)	0.38 (0.14 to 0.93)	0.32 (0.22 to 0.42)	<0.001
Republic of Namibia	0 (0 to 0)	0.11 (0.05 to 0.18)	1.59 (0.8 to 2.87)	0.12 (0.06 to 0.22)	0.72 (0.55 to 0.89)	<0.001
Republic of Nauru	0 (0 to 0)	0.23 (0.1 to 0.46)	0.01 (0.01 to 0.03)	0.23 (0.1 to 0.44)	-0.03 (-0.14 to 0.07)	0.537
Republic of Nicaragua	0 (0 to 0)	0.06 (0.04 to 0.09)	3.94 (2.15 to 6.84)	0.11 (0.06 to 0.19)	2.99 (2.75 to 3.23)	<0.001
Republic of Niue	0 (0 to 0)	0.16 (0.07 to 0.3)	0 (0 to 0)	0.17 (0.07 to 0.34)	0.18 (0.03 to 0.33)	0.02

Republic of Palau	0 (0 to 0)	0.35 (0.16 to 0.7)	0.04 (0.02 to 0.08)	0.48 (0.23 to 0.87)	1.46 (1.02 to 1.9)	<0.001
Republic of Panama	0 (0 to 0)	0.06 (0.04 to 0.09)	2.7 (1.65 to 4.05)	0.12 (0.08 to 0.19)	3.61 (3.47 to 3.75)	<0.001
Republic of Paraguay	0 (0 to 0)	0.09 (0.06 to 0.14)	5.53 (2.96 to 9.28)	0.14 (0.08 to 0.24)	2.04 (1.93 to 2.15)	<0.001
Republic of Peru	10 (0 to 10)	0.06 (0.04 to 0.1)	16.74 (8.99 to 28.9)	0.09 (0.05 to 0.15)	1.58 (1.46 to 1.7)	<0.001
Republic of Poland	10 (10 to 10)	0.04 (0.03 to 0.04)	24.18 (19.25 to 29.32)	0.13 (0.11 to 0.16)	6.58 (6.3 to 6.85)	<0.001
Republic of Rwanda	10 (10 to 20)	0.27 (0.14 to 0.48)	9.73 (4.62 to 17.67)	0.14 (0.07 to 0.26)	-3.05 (-3.26 to -2.85)	<0.001
Republic of San Marino	0 (0 to 0)	0.12 (0.07 to 0.18)	0.02 (0.01 to 0.05)	0.18 (0.08 to 0.34)	2.13 (2.02 to 2.24)	<0.001
Republic of Senegal	10 (10 to 20)	0.24 (0.11 to 0.45)	14.84 (7.73 to 27.03)	0.19 (0.1 to 0.35)	-1.06 (-1.11 to -1.01)	<0.001
Republic of Serbia	10 (10 to 20)	0.3 (0.18 to 0.47)	9.66 (5.43 to 15.93)	0.23 (0.13 to 0.38)	-1.33 (-1.51 to -1.16)	<0.001
Republic of Seychelles	0 (0 to 0)	0.19 (0.1 to 0.32)	0.13 (0.07 to 0.22)	0.24 (0.12 to 0.4)	0.98 (0.82 to 1.14)	<0.001
Republic of Sierra Leone	10 (0 to 20)	0.56 (0.18 to 1.15)	11.78 (5.49 to 22.36)	0.26 (0.12 to 0.5)	-3.58 (-3.85 to -3.31)	<0.001
Republic of Singapore	0 (0 to 0)	0.09 (0.05 to 0.16)	1.54 (0.8 to 2.76)	0.05 (0.03 to 0.09)	-2.86 (-3.52 to -2.18)	<0.001
Republic of Slovenia	0 (0 to 0)	0.25 (0.15 to 0.39)	1.51 (0.83 to 2.37)	0.17 (0.1 to 0.27)	-1.83 (-1.93 to -1.72)	<0.001
Republic of South Africa	120 (90 to 150)	0.48 (0.36 to 0.62)	110.32 (79.56 to 147.64)	0.35 (0.26 to 0.47)	-1.53 (-1.85 to -1.2)	<0.001
Republic of South Sudan	0 (0 to 10)	0.12 (0.05 to 0.22)	6.72 (2.99 to 13.58)	0.15 (0.07 to 0.31)	1.32 (1.15 to 1.48)	<0.001
Republic of Sudan	0 (0 to 10)	0.04 (0.02 to 0.08)	8.39 (3.78 to 15.83)	0.04 (0.02 to 0.07)	-0.15 (-0.3 to 0.01)	0.059
Republic of Suriname	0 (0 to 0)	0.11 (0.06 to 0.17)	0.44 (0.24 to 0.8)	0.15 (0.08 to 0.28)	1.83 (1.67 to 1.98)	<0.001
Republic of Tajikistan	0 (0 to 10)	0.14 (0.07 to 0.26)	6.44 (3.3 to 11.82)	0.13 (0.06 to 0.23)	-0.67 (-0.72 to -0.62)	<0.001
Republic of the Congo	0 (0 to 10)	0.24 (0.1 to 0.51)	6.5 (2.3 to 14.12)	0.23 (0.08 to 0.5)	-0.24 (-0.35 to -0.12)	<0.001
Republic of the Gambia	10 (0 to 10)	0.98 (0.48 to 1.72)	12.29 (5.67 to 23.72)	1.04 (0.48 to 2)	0.23 (0.07 to 0.38)	0.003
Republic of the Marshall Islands	0 (0 to 0)	0.07 (0.03 to 0.14)	0.03 (0.01 to 0.07)	0.1 (0.04 to 0.24)	1.6 (1.38 to 1.82)	<0.001
Republic of the Niger	20 (10 to 40)	0.35 (0.14 to 0.76)	17.9 (8.1 to 39.08)	0.17 (0.08 to 0.38)	-3.36 (-3.41 to -3.32)	<0.001
Republic of the Philippines	150 (110 to 200)	0.37 (0.27 to 0.5)	196.94 (137.37 to 277.36)	0.33 (0.23 to 0.46)	-0.66 (-0.82 to -0.5)	<0.001
Republic of the Union of Myanmar	20 (10 to 50)	0.08 (0.03 to 0.2)	30.76 (11.71 to 70.9)	0.1 (0.04 to 0.24)	1.09 (1.05 to 1.13)	<0.001
Republic of Trinidad and Tobago	0 (0 to 0)	0.1 (0.06 to 0.16)	0.98 (0.56 to 1.54)	0.14 (0.08 to 0.22)	1.57 (1.5 to 1.64)	<0.001
Republic of Tunisia	0 (0 to 0)	0.03 (0.01 to 0.05)	2.7 (1.34 to 5.2)	0.04 (0.02 to 0.09)	2.57 (2.37 to 2.78)	<0.001
Republic of Turkey	10 (10 to 20)	0.03 (0.02 to 0.06)	18.09 (9.27 to 31.47)	0.04 (0.02 to 0.07)	0.93 (0.83 to 1.03)	<0.001
Republic of Uganda	30 (20 to 50)	0.33 (0.18 to 0.52)	59.2 (29.94 to 105.42)	0.29 (0.15 to 0.52)	-0.47 (-0.64 to -0.31)	<0.001
Republic of Uzbekistan	20 (10 to 30)	0.13 (0.07 to 0.22)	30.26 (16.89 to 49.92)	0.17 (0.09 to 0.28)	1.26 (1.11 to 1.4)	<0.001
Republic of Vanuatu	0 (0 to 0)	0.07 (0.03 to 0.15)	0.13 (0.06 to 0.26)	0.08 (0.04 to 0.17)	0.56 (0.45 to 0.67)	<0.001
Republic of Yemen	0 (0 to 0)	0.02 (0.01 to 0.04)	2.27 (0.87 to 4.99)	0.01 (0.01 to 0.03)	-0.48 (-0.62 to -0.35)	<0.001
Republic of Zambia	20 (10 to 40)	0.4 (0.17 to 0.79)	13.75 (3.88 to 42.97)	0.14 (0.04 to 0.44)	-4.92 (-5.2 to -4.64)	<0.001
Republic of Zimbabwe	30 (10 to 50)	0.47 (0.23 to 0.81)	30.04 (14.12 to 55.71)	0.39 (0.18 to 0.72)	-0.93 (-1.09 to -0.76)	<0.001
Romania	10 (10 to 20)	0.13 (0.08 to 0.19)	17.76 (10.94 to 26.14)	0.21 (0.13 to 0.31)	2.6 (2.4 to 2.81)	<0.001
Russian Federation	130 (110 to 160)	0.16 (0.13 to 0.19)	154.77 (122.88 to 189.2)	0.23 (0.18 to 0.28)	1.78 (1.39 to 2.17)	<0.001
Saint Kitts and Nevis	0 (0 to 0)	0.14 (0.08 to 0.21)	0.04 (0.02 to 0.07)	0.12 (0.07 to 0.21)	-0.58 (-0.89 to -0.26)	<0.001

Saint Lucia	0 (0 to 0)	0.12 (0.08 to 0.18)	0.14 (0.08 to 0.21)	0.15 (0.09 to 0.23)	0.8 (0.46 to 1.14)	<0.001
Saint Vincent and the Grenadines	0 (0 to 0)	0.14 (0.09 to 0.21)	0.13 (0.08 to 0.2)	0.24 (0.15 to 0.35)	2.57 (2.43 to 2.71)	<0.001
Slovak Republic	10 (10 to 10)	0.32 (0.2 to 0.48)	7.34 (3.82 to 12.66)	0.28 (0.15 to 0.49)	-0.62 (-0.71 to -0.52)	<0.001
Socialist Republic of Viet Nam	190 (90 to 330)	0.43 (0.22 to 0.75)	407.97 (193 to 767.43)	0.78 (0.37 to 1.47)	2.88 (2.83 to 2.92)	<0.001
Solomon Islands	0 (0 to 0)	0.12 (0.05 to 0.25)	0.47 (0.2 to 0.95)	0.14 (0.06 to 0.28)	0.66 (0.51 to 0.82)	<0.001
State of Eritrea	0 (0 to 0)	0.14 (0.06 to 0.26)	3.54 (1.45 to 6.96)	0.1 (0.04 to 0.2)	-1.4 (-1.52 to -1.27)	<0.001
State of Israel	0 (0 to 0)	0.08 (0.05 to 0.13)	3.76 (2.22 to 5.88)	0.08 (0.05 to 0.13)	0.2 (0.13 to 0.27)	<0.001
State of Kuwait	0 (0 to 0)	0.02 (0.01 to 0.03)	0.29 (0.15 to 0.51)	0.01 (0 to 0.02)	-3.29 (-3.67 to -2.9)	<0.001
State of Libya	0 (0 to 0)	0.04 (0.02 to 0.08)	4.4 (1.93 to 8.48)	0.11 (0.05 to 0.21)	4.4 (4.15 to 4.64)	<0.001
State of Qatar	0 (0 to 0)	0.1 (0.05 to 0.19)	1.56 (0.77 to 2.82)	0.07 (0.03 to 0.13)	-1.69 (-1.94 to -1.44)	<0.001
Sultanate of Oman	0 (0 to 0)	0.03 (0.02 to 0.06)	1.15 (0.53 to 2.22)	0.04 (0.02 to 0.07)	0.68 (0.55 to 0.82)	<0.001
Swiss Confederation	10 (10 to 20)	0.34 (0.23 to 0.48)	7.1 (4.34 to 10.83)	0.18 (0.11 to 0.27)	-3.08 (-3.3 to -2.86)	<0.001
Syrian Arab Republic	0 (0 to 10)	0.04 (0.02 to 0.08)	3.44 (1.58 to 6.88)	0.05 (0.02 to 0.1)	0.34 (-0.55 to 1.23)	0.456
Taiwan (Province of China)	30 (20 to 50)	0.24 (0.13 to 0.4)	20.66 (10.78 to 37.49)	0.18 (0.09 to 0.33)	-1.37 (-1.75 to -0.99)	<0.001
Togolese Republic	10 (0 to 10)	0.27 (0.12 to 0.55)	9.61 (4.59 to 20.07)	0.23 (0.11 to 0.48)	-0.69 (-0.89 to -0.5)	<0.001
Tokelau	0 (0 to 0)	0.08 (0.04 to 0.19)	0 (0 to 0)	0.13 (0.06 to 0.27)	2.08 (1.67 to 2.49)	<0.001
Turkmenistan	0 (0 to 10)	0.17 (0.1 to 0.27)	6.41 (3.42 to 10.98)	0.24 (0.13 to 0.41)	1.5 (1.38 to 1.61)	<0.001
Tuvalu	0 (0 to 0)	0.11 (0.05 to 0.22)	0.01 (0 to 0.01)	0.12 (0.05 to 0.22)	0.14 (-0.06 to 0.34)	0.163
Ukraine	50 (40 to 60)	0.19 (0.14 to 0.25)	26.31 (16.36 to 39.03)	0.13 (0.08 to 0.19)	-1.88 (-2.61 to -1.14)	<0.001
Union of the Comoros	0 (0 to 0)	0.11 (0.05 to 0.19)	0.46 (0.21 to 0.87)	0.12 (0.05 to 0.22)	0.53 (0.35 to 0.71)	<0.001
United Arab Emirates	0 (0 to 0)	0.07 (0.03 to 0.13)	8.31 (3.92 to 15.68)	0.12 (0.06 to 0.23)	2.68 (2.31 to 3.05)	<0.001
United Kingdom of Great Britain and Northern Ireland	60 (50 to 70)	0.2 (0.16 to 0.24)	140.24 (110.64 to 170.15)	0.46 (0.36 to 0.56)	4.07 (3.93 to 4.21)	<0.001
United Mexican States	30 (30 to 40)	0.06 (0.05 to 0.08)	77.41 (58.86 to 97.23)	0.11 (0.09 to 0.14)	2.73 (2.68 to 2.78)	<0.001
United Republic of Tanzania	30 (10 to 50)	0.18 (0.09 to 0.33)	42.26 (19.72 to 78.74)	0.15 (0.07 to 0.28)	-0.82 (-0.87 to -0.77)	<0.001
United States of America	510 (430 to 600)	0.35 (0.3 to 0.42)	435.1 (354.78 to 523.77)	0.29 (0.23 to 0.34)	-0.96 (-1.23 to -0.69)	<0.001
United States Virgin Islands	0 (0 to 0)	0.13 (0.07 to 0.21)	0.07 (0.03 to 0.12)	0.2 (0.1 to 0.35)	1.94 (1.82 to 2.06)	<0.001

Abbreviation: CI: confidence interval; UI: uncertainty interval

Supplementary Table S2 Prevalence and age-standardized prevalence rates of patients with alcohol-associated cirrhosis and chronic liver disease in young adults in 2021 and changes from 2000 to 2021

	2021 Prevalence (95% UI)	2021 ASPR (95% UI)	2000 to 2021 APC (95% CI)	***p***
Overall	1018270 (784660 to 1290080)	25.79 (19.87 to 32.67)	-0.13 (-0.17 to -0.1)	<0.001
**Sex**				
Female	213550 (158230 to 285720)	10.96 (8.12 to 14.66)	-0.52 (-0.67 to -0.37)	<0.001
Male	804730 (623860 to 1016090)	40.24 (31.2 to 50.81)	-0.04 (-0.06 to -0.01)	0.003
**Region**				
Africa	63500 (43590 to 87900)	11.35 (7.79 to 15.71)	-0.31 (-0.4 to -0.22)	<0.001
Eastern Mediterranean	16820 (11740 to 23430)	4.2 (2.93 to 5.86)	0.21 (0.16 to 0.27)	<0.001
Europe	279100 (216110 to 359190)	65.29 (50.55 to 84.02)	0.17 (0.02 to 0.32)	0.026
Region of the Americas	154220 (117590 to 196370)	30.07 (22.93 to 38.29)	-0.87 (-0.95 to -0.79)	<0.001
Southeast Asia	400910 (305340 to 502770)	35.63 (27.13 to 44.68)	0.45 (0.42 to 0.49)	<0.001
Western Pacific	96530 (71650 to 127980)	10.64 (7.9 to 14.11)	0.56 (0.5 to 0.61)	<0.001
**SDI**				
Low SDI	87280 (64320 to 115250)	16.09 (11.86 to 21.25)	-0.53 (-0.55 to -0.51)	<0.001
Low-middle SDI	271840 (205360 to 347190)	26.75 (20.21 to 34.16)	0.25 (0.23 to 0.28)	<0.001
Middle SDI	290520 (223460 to 370680)	23.15 (17.8 to 29.53)	0.58 (0.53 to 0.63)	<0.001
High-middle SDI	234540 (180350 to 303970)	37.25 (28.65 to 48.28)	0.42 (0.27 to 0.57)	<0.001
High SDI	133170 (101480 to 168960)	26.52 (20.21 to 33.64)	-1.52 (-1.66 to -1.38)	<0.001

Abbreviation: APC: annual percent change; ASPR: age-standardized prevalence rate; CI: confidence interval: SDI: sociodemographic index: UI: uncertainty interval
